# Early triadic interactions in the first year of life: a systematic review on object-mediated shared encounters

**DOI:** 10.3389/fpsyg.2023.1205973

**Published:** 2023-08-17

**Authors:** Ana Mendoza-García, Ana Moreno-Núñez

**Affiliations:** Departamento de Psicología Evolutiva y de la Educación, Universidad Autónoma de Madrid, Madrid, Spain

**Keywords:** triadic interactions, referential communication, joint action, materiality, early development

## Abstract

Infants’ early interactions with adults and everyday objects are key to socio-communicative development, but their emergence and development are still under debate. Aiming at describing the diversity of theoretical and methodological approaches on triadicity during the first year of life, we conducted a systematic and qualitative review of recent literature. Following PRISMA 2020 guidelines, we explored the scientific production of recent decades on triadic interactions up to 12 months of age. We initially screened 1943 items from which we obtained a final sample of 51 publications. Studies are usually conducted in laboratory settings, while ecological research is becoming increasingly common, especially in home settings. According to a thematic analysis of the data, we discussed the different perspectives on the origin and conceptualization of triadic interactions, and how they contribute to structuring and facilitating other developmental phenomena, such as the children’s communicative gestures and uses of objects. Prior to the origin of intentional communication, adults facilitate early forms of triadicity based on fostering opportunities for infants’ communication and engagement with both adults and materiality. However, there is a need for further research that explore the potential of early triadic interactions for parenting and early childhood education practises.

## 1. Introduction

The first social experiences of infants take place, from the first months of life, through interactions with their caregivers and with everyday objects (Moreno-Núñez et al., 2021a). This is consistent with the cultural-historical approach and, more specifically, with the postulates of activity theory developed from Vygotskian proposals. From this perspective, individuals act collectively based on shared activities and communicative exchanges that allow us to appropriate cultural tools (Vygotsky, 1978; Foot, 2014). According to this, social interactions, especially with adults, are essential during early childhood as they favour an inter-communicative space of mediation and collaboration that facilitates the child’s learning and development (Vygotsky, 1984; Veraksa and Veraksa, 2018).

Adult mediation refers to the guided process of equipping children, through shared activities, with the psychological tools necessary to operate in a specific sociocultural context. It enables children to progressively internalise their learning and, consequently, to develop in the world in an increasingly autonomous way (Vygotsky, 1934/1986). Throughout the first year, infants’ relationship with their environment is modified by the intervention of the adult (Vygotsky, 1932/1996; McCune and Zlatev, 2015), who responds to their first signals and attempts to communicate while sharing bouts of attention and action on specific objects or events (Bakeman and Adamson, 1984; Solovieva and Quintanar, 2021). This process does not, therefore, occur in a vacuum, but within the framework of early interactions that take place in a given socio-material context. In these interactions, objects are incorporated not as mere material realities, but as communicative vehicles that encourage joint action based on their social and cultural properties (Rodríguez, 2012; Burner and Svendsen, 2020). Thus, it is in these interactions where the conventional meanings and uses of objects are transmitted (Rodríguez, 2006; Solovieva et al., 2020). This enables progressive sophistication in infants’ actions, by turning these into social acts directed to, and coordinated with, the other person (Carpendale et al., 2021).

The role of these triadic encounters (adult-infant-object) in socio-communicative development has been the focus of much debate, especially over their origin. Traditionally, it has been considered that babies relate first to adults, during the first months of life, and somewhat later, also to objects. This has given rise to the differentiation between primary intersubjectivity, referring to early dyadic exchanges between adults and infants, and secondary intersubjectivity, in which interactions incorporate external referents, acquiring a triadic character (Trevarthen and Hubley, 1978; Carpenter et al., 1998; Tomasello et al., 2005; Striano and Reid, 2006). The transition from one stage to the other is usually considered to take place at around nine months of age, when the infants’ first intentional communicative acts towards the adult emerge in what Tomasello (2013) called the “nine-month revolution.” From then on, the complexity of the infants’ socio-communicative behaviours increases, as they can share experiences with others through, for example, the use of gestures and the diversification of communicative functions (Bates et al., 1975).

However, more recent work questions whether the emergence of infants’ first triadic experiences is limited to the end of the first year (Rodríguez, 2006). Some authors have characterised this process as a continuous and gradual development, mediated by the adult from birth (Reddy, 2010). In this sense, early communication would develop from the beginning of life according to interactional dynamic processes (Fogel and Thelen, 1987; Fogel, 1993; Thelen and Smith, 1994) in which what is shared is not transmitted unidirectionally, from one mind to another, but is jointly created between adult and child (Fogel, 1993; Trevarthen and Reddy, 2017) or within the family dynamics with more than one primary caregiver (Fivaz-Depeursinge and Corboz-Warnery, 1999; León and Olhaberry, 2020). Communicative intentionality, therefore, is gradually co-constructed and integrated as part of the infant’s repertoire of skills, thanks to the adult-mediated encounters that take place over time (Rączaszek-Leonardi et al., 2019) and in which they often incorporate objects to communicate about—and through—the material world (Rodríguez, 2012).

Precisely, the lack of consideration given to objects in early interactions is another of the problems underlying their conceptualisation. From classical positions, triadic interactions with objects are based on episodes of joint attention, which can be understood from different perspectives (Gabouer and Bortfeld, 2021). On the one hand, from an associative perspective they would depend on the infant’s visual orientation system and its ability to follow the gaze of the other (Butterworth, 1991), which is considered to occur in the absence of interactive processes from six to 18 months of age (Butterworth and Jarrett, 1991; Corkum and Moore, 1998). On the other hand, from a social perspective, this highlights the coincidence of gazes between adults and infants on a referent (e.g., objects) in the framework of a dynamic interaction process that begins early in the first year of life (Striano and Stahl, 2005) but continues to develop throughout the second year (Tomasello and Farrar, 1986). Indeed, some studies emphasise the attentional, communicative, and co-regulatory processes between adults and infants in relation to an object of mutual interest, that unfold between 6 months and 1.5 years old. In this way, they distinguish between “coordinated joint engagement,” which refers to the multimodal components that occur in intersubjective episodes (e.g., smiles or gestures), and “passive joint engagement,” which would be limited to the coincidence of attentional focus on the same object (Bakeman and Adamson, 1984).

While the joint attention paradigm has occupied a central role in research over the last few decades, recent studies invite us to modify the analytical prism based on the growing interest in the multimodal components that underpin these adult-infant encounters (Yu and Smith, 2016, 2017). For example, adults tend to accompany episodes of joint attention by combining verbal input and tactile stimulation towards the infant (Suárez-Rivera et al., 2019). This could be associated with a longer duration of the infant’s gaze towards the object, compared to non-multimodal episodes. This evidence has called into question the study of joint attention as an isolated process, as it could be part of a conglomerate of cognitive, social, and communicative phenomena (Siposova and Carpenter, 2019; Jacobson and Degotardi, 2022) that take place in joint action dynamics between the infant and others (Reddy, 2005). In this sense, adopting an interactive and multimodal perspective would make it possible to emphasise not only attentional aspects but also other elements of the interaction such as emotion, touch, or use of objects (De Barbaro et al., 2013; Rossmanith et al., 2014; Moreno-Núñez et al., 2017).

The lack of an established consensus around triadic interactions, such as how and when they emerge, or the particular characteristics that arise from their conceptualisation has left an open theoretical and methodological debate. As such, there may be reason to identify those inconclusive areas and to explore the different perspectives on their origin, development, and nature of triadic interactions. To this end, we conducted a systematic and qualitative review that explored the scientific production of the last decades and its main findings, discussing their potential implications for the study of early development and early childhood education and care. In doing so, we can inform further empirical exploration of this phenomena.

## 2. Methods

Following the recommendations of the PRISMA 2020 statement (Page et al., 2021), an updated version of PRISMA 2009 (Moher et al., 2009), we conducted a comprehensive literature search in eight databases: Web of Science, Scopus, ProQuest, PubMed, ScienceDirect, PsycINFO, Dialnet, and Dimensions. To do so, we started with four keywords, in English and Spanish, which were combined with Boolean operators according to the following search equation: (“triadic interactions” OR “joint engagement”) AND object* AND child*. In addition to the articles recovered from the databases, other relevant studies, retrieved from the references cited in other research, were also included.

**Figure 1:**
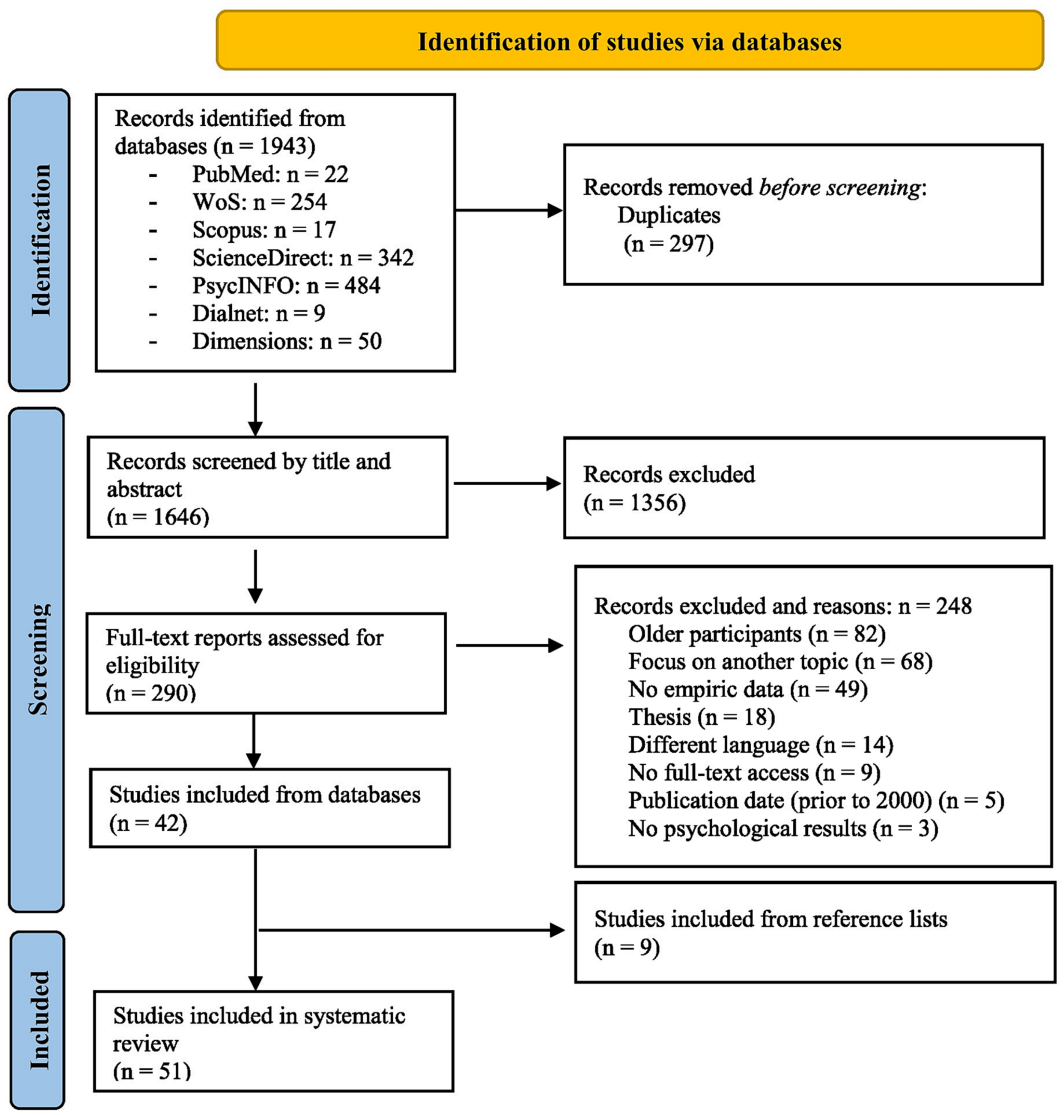
PRISMA Flow chart.

The PRISMA flow chart (Figure 1) illustrates the study selection process performed for this systematic review. The total number of results obtained in the initial search was 1,943 articles, of which 297 duplicates were eliminated. We then collected general information on each study (author, year of publication, title, source database and abstract) and reviewed their appropriateness to the study topic, based on the title and abstract. This resulted in the exclusion of 1,356 articles that were not related to our objectives, as they focussed, for example, on parental mental health, family interventions or research with non-human primates. From the resulting database (*n* = 290), full texts were analysed and screened according to the following eligibility criteria:

- *Publication type*: empirical articles published in peer-reviewed scientific journals were selected. Doctoral theses, book chapters, monographs, theoretical articles, and reviews were excluded.
- *Publication area*: we included studies that provided results in psychological terms (human development or behaviour).
- *Aim of study*: publications selected were those that explored triadic interactions between adults, objects, and infants during the first months of life. Articles on related topics (joint attention) were included if they referred to the ‘triadicity’ of the interactions.
- *Age of participants*: given our interest in early interactions, we have prioritised the inclusion of studies focussing on the first year of life. Nevertheless, longitudinal studies where data collection was extended up to 18 months of age of the participants were also considered.
- *Language*: only articles published in English or Spanish were included.
- *Date of publication*: to provide an updated analysis, only studies published between 2000 and 2022 were considered.
- *Access to full text*: articles for which it has not been possible to access the full text for review were also excluded.

During the decision-making process, discrepancies regarding the inclusion of certain articles were resolved by consensus among the investigators. The screening process resulted in a final sample of 51 publications, which were subjected to a methodological quality analysis using QualSyst (Kmet et al., 2004). This tool provides checklists adapted to a wide range of methodologies and topics that allow the critical evaluation of qualitative and quantitative scientific papers. On this basis, it provides an estimate of the methodological quality of studies as a result of a score between 0 and 1, with 1 being the highest possible quality score. It also proposes different cut-off points for deciding on the exclusion of studies, ranging from 0.55 to 0.75. All the studies included in the final sample exceed the highest cut-off point of 0.75 (M = 0.925; SD = 0.06).

## 3. Results

For the analysis, we first explored the distribution of the scientific production included in the sample in terms of its population characteristics (where the studies were carried out and the context in which the data were collected) and methodological characteristics (type of study, type of design, type of interaction task, and data analysis strategy). Secondly, we carried out a thematic analysis of the results, based on the discussion of findings related to the origin, development, and educational implications of triadic interactions.

### 3.1. Description of recent scientific production

**Figure 2:**
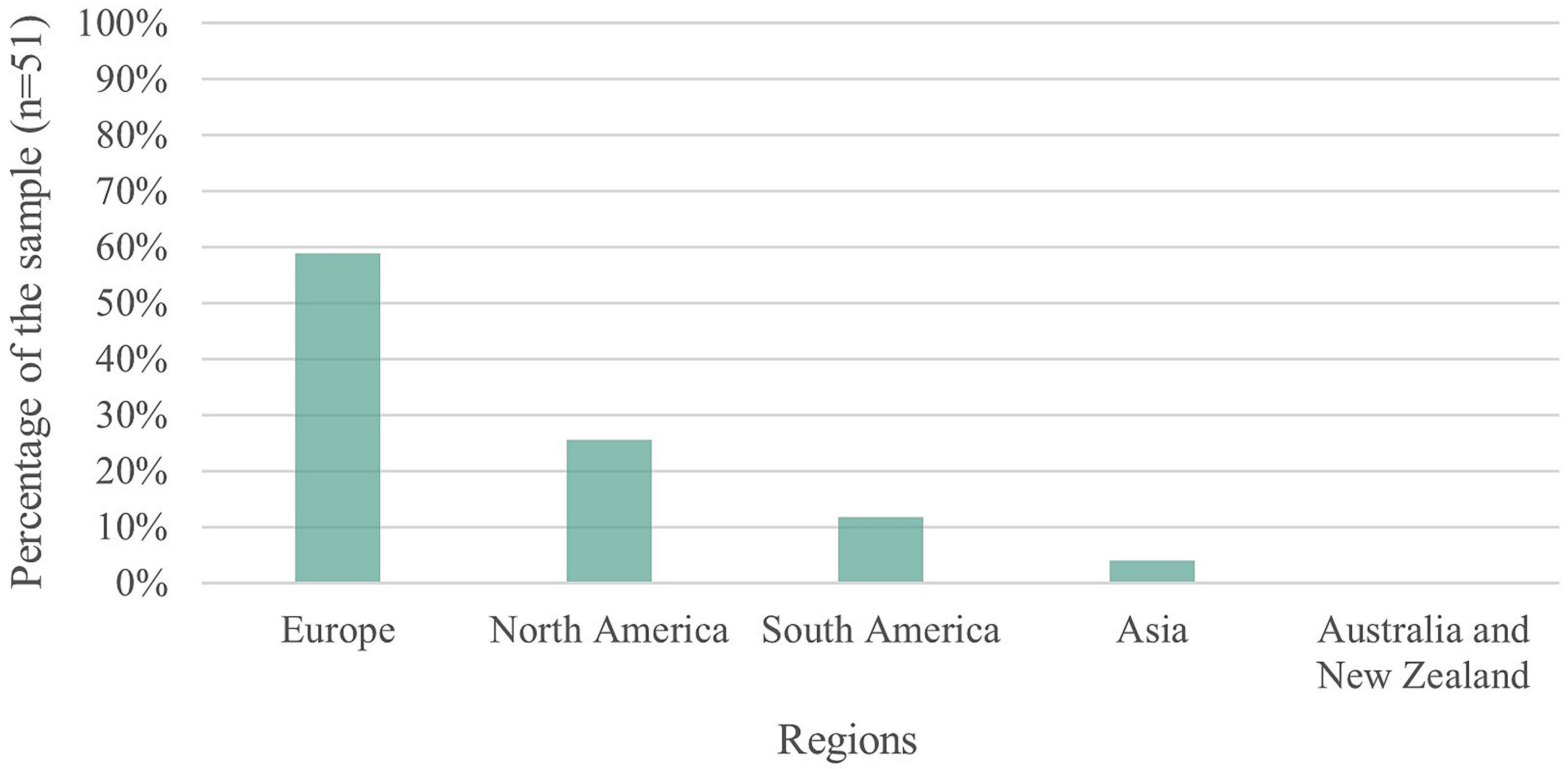
Sample distribution according to the regions where the studies were conducted.

When analysing the data on the location where the studies were conducted (Figure 2), we observed that studies run in European countries (59%) and North America (25%) predominated. Nonetheless, probably since our review also included texts in Spanish, the sample includes a considerable percentage of studies run in Latin American countries (12%). The limited share of research run in Asian contexts was striking (4%), with only two studies run in Japan and India, respectively. We did not find any work on this subject based on data collected in Australia or New Zealand.

**Figure 3:**
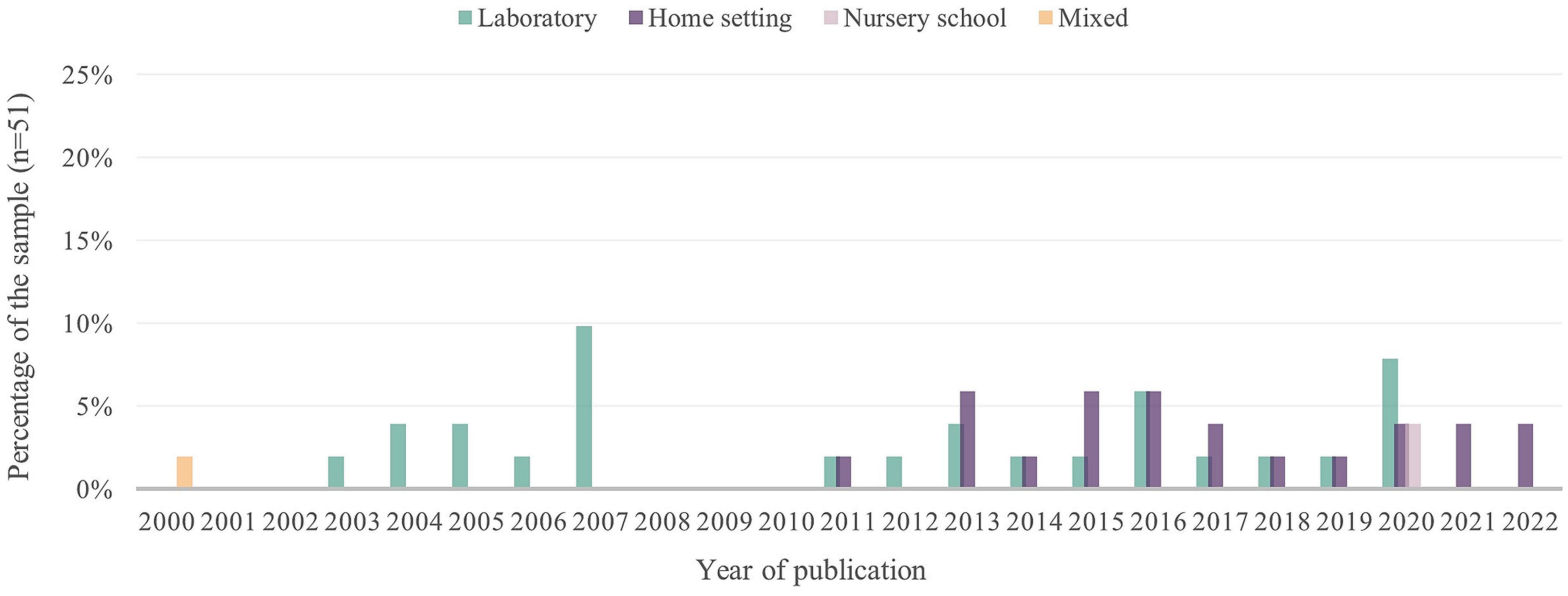
Sample distribution by year of publication according to the settings in which the interactions were observed.

We also looked at recent trends in the study of triadic interactions in different contexts (Figure 3), as these could lead to different ways of interacting with the environment. To this end, we defined four categories that classify the interactions according to the degree of control of variables: laboratory, home, nursery school and mixed categories (i.e., studies that combined two or more of the above). The laboratory was and is the predominant setting for data collection in most of the research analysed (53%). However, there has been a recent increase in the number of studies using data collected in naturalistic settings, especially the home (41%). This includes studies in which the interactions take place between the child and their mother/father in a familiar environment (e.g., their own home or school). Nonetheless, other everyday interactions in the infants’ life, such as those with educators and peers at nursery school, are still underrepresented in the scientific literature (4%). Finally, although we found only one such study (2% of the sample), it is important to note that we were able to find mixed studies that combined data from more than one of these contexts, for example, the home and the laboratory (Danis et al., 2000). However, this is not an alternative that has been maintained over time, favouring studies focussing on one or the other context.

Finally, Table 1 shows the distribution of the sample in terms of three elements: first, the type of study; second, the type of empirical design; and third, the data analysis strategy (Montero and León, 2002). For comprehensiveness purposes, we also included the type of interaction task used in the studies in our sample. Regarding the type of study, the sample comprises mainly descriptive (71%) and experimental (29%) research. In relation to the type of empirical design, we observed a more homogeneous distribution between longitudinal (55%) and cross-sectional designs (43%), while pre-post test designs in this area are scarce (2%). Studies were most frequently based on free-play interactions between adults (both researchers and parents) and babies (74.5%), followed by experimental interactions (17.5%) aimed at eliciting certain behaviours in infants. We found very few studies based on everyday routines at home such us feeding activities (4%) or teacher-led interactions in nursery schools (4%). Although not all studies provided data on the type of objects utilised, the vast majority of studies in our sample used toys (including musical toys and books) and artifacts (e.g., a spoon) to promote interaction. Finally, according to the data analysis strategy, there is a significant predominance of quantitative studies (78%), compared to qualitative (10%) or mixed methods (12%).

**Table 1:** Frequency and percentage of studies in the sample (*n* = 51) by type of study, type of design, type of interaction task, and data analysis strategy.

		No. of studies	% of sample
Study type	Descriptive	36	71%
	Experimental	15	29%
Design type	Longitudinal	28	55%
	Cross-sectional	22	43%
	Pre-post test	1	2%
Interaction task type	Free play	38	74.5%
	Experimental	9	17.5%
	Teacher-led	2	4%
	Everyday routine	2	4%
Data analysis strategy	Quantitative	40	78%
	Qualitative	5	10%
	Mixed-methods	6	12%

### 3.2. Thematic analysis of the data

The second part of this study consisted of a thematic analysis of the data (Braun and Clarke, 2006), for which we organised the results along four lines of discussion: (1) the phenomena associated with the beginnings of triadicity, (2) the origin of early triadic behaviours, (3) the process of co-construction of adult-infant interaction, and (4) the function of triadic interactions as a shared learning framework.

#### 3.2.1. The first triadic encounters: from joint attention to joint action

One of the most studied phenomena on the origin of triadicity in early infancy is joint attention, which is an inherently socio-communicative process based on social cognition and communicative intentionality. While much research has confined its empirical study to aspects related to the infant’s visual orientation system, for example, through measures such as gaze fixation and/or visual trajectory tracking between three and 12 months of age (Cleveland and Striano, 2007; Parise et al., 2007; Tremblay and Rovira, 2007; Gattis et al., 2020), other work proposes that joint attention should be described on the basis of infant and adult socio-communicative skills that mediate interactions with objects throughout the entire first year of life (Striano and Bertin, 2005; Striano and Stahl, 2005; Striano et al., 2007; Osório et al., 2011). Among these, gaze following seems to emerge as an essential component from the first months after birth (Perra and Gattis, 2012), although it is not the only one.

There are more behaviours that make it possible to sustain and participate in the interaction, for example, the visual following of adults’ pointing gestures that arise around three to 4 months of age, and object manipulations in the last third of the first year (Amano et al., 2004; Flom et al., 2004). Although these studies have made important contributions to the study of early perceptual-attentional development, their point of view is limited in that they do not incorporate other variables of the psychological relationships between adults, objects, and infants, such as their multimodal characteristics or the construction of shared meanings in the interaction.

This problem, in part, derives from the lack of attention from this approach to the role played by referents in triadic interactions. Objects and their cultural norms of use favour and catalyse interactions by constituting a shared referent and are, therefore, essential when analysing triadic exchanges, especially at a very early age (Dimitrova and Moro, 2013; Palacios and Rodríguez, 2015; Dupertuis and Moro, 2016; Moreno-Núñez et al., 2017; Alessandroni et al., 2020; Vietri et al., 2021). In a study based on shared reading situations, Rossmanith et al. (2014) described how adults, from the age of 3 months of the baby, turn the object into an element both to be attended to (for example, by showing the book and using sounds, vocalisations, and verbalisations) and acted upon together (turning pages, opening fold-out flaps, touching different textures, or pointing to pictures). From this point of view, attention and joint action are two inseparable parts of complex, multimodal, socio-communicative exchanges, in which objects act as communicative vehicles between adults and babies (Moreno-Núñez et al., 2021b). At the beginning, it is the adult who endows these encounters with a triadic character, by progressively facilitating the infants’ learning about how to relate to and operate in the world.

#### 3.2.2. The origins of the development of triadicity

Given the different perspectives on when the first triadic interactions emerge, we considered that this could be reflected in the designs represented in the sample. For example, considering that the interaction is only triadic from the age of 9 months onwards, this might have an impact on the age selection of participants, resulting in a lower volume of evidence in earlier months. To this end, we explored the distribution of studies according to the ages analysed (in months). Since some studies addressed a particular age range, each age was considered as a non-mutually exclusive category. Thus, Table 2 shows that there is a higher proportion of studies in the sample as the infants’ age increases, especially during the last third of the first year of life, where 53% of the total observations were concentrated (Flom et al., 2004; Abels and Hutman, 2015; Abels, 2020). These studies provide relevant information from the time when infants already possess some skill in triadic communication (Parise et al., 2007; Osório et al., 2011; Mendive et al., 2013). Nevertheless, by focussing only on when infants acquire this achievement, they leave unresolved how these skills are developed at earlier stages.

**Table 2:** Frequency and percentage of studies in the sample (*n* = 51) according to participants’ age in months.

	Months of age												
	1	2	3	4	5	6	7	8	9	10	11	12	> 12
No. studies	1	6	12	17	15	18	16	19	29	25	24	29	15
% sample	2%	12%	24%	33%	29%	35%	31%	37%	57%	49%	47%	57%	29%

Despite the above, in recent decades there has been an increase in studies that specifically focus on early social-communicative skills (Amano et al., 2004; Striano and Bertin, 2005; Striano et al., 2007; Tremblay and Rovira, 2007). For example, Striano and Stahl (2005) already suggested that triadic skills develop progressively over the first months of life, based on a comparative study on the duration of infants’ gaze. Two experimental conditions were designed to run this: in the first, the adult presented an object to the infant accompanied by affectionate comments, while coordinating visual attention between the object and the infant; in the second, the adult presented the object in the same way, but this time only directing the gaze towards the object. They found that, already at 3 months, infants’ gaze duration was longer in the first experimental condition, which may indicate some early sensitivity to relevant communicative cues, such as eye contact with the adult in relation to the object.

This highlights the role played by the adult in the infant’s daily exchanges and routines in the environment and, specifically, in relation to the material world. Recently, other studies suggested that the adult actively involves the infant and the object in the same communicative act from the first months of life (De Barbaro et al., 2013, 2016; Rossmanith et al., 2014; Moreno-Núñez et al., 2017), producing early triadic interactions even when infants are not yet able to initiate them on their own (Moreno-Núñez et al., 2017). From birth, adults and infants habitually engage in interactions based on iterative actions with objects, which provide a defined and facilitating structure (Moreno-Núñez et al., 2015; Alessandroni et al., 2020). The reiterative and organised environment in which the first triadic interactions take place also favours attunement and understanding actions between infants and adults (Dimitrova et al., 2015). Redundancies created, for example, through stimulation by touch, may occur in response to situations where the affective mediation of the adult becomes more necessary. This may occur, either because of the very young age of infants (Alessandroni et al., 2020), or the difficulty of the task, where adults accompany infants in their attempts (Serra et al., 2020).

#### 3.2.3. Co-constructing the interaction: the role of the adult in the infant’s developing agency

In line with this idea of early triadic interactions, i.e., before the age of 9 months, more research seeks to describe the role of the adult in establishing the first shared references jointly with the child (Danis et al., 2000; Hobson et al., 2004; Moreno-Núñez et al., 2017). This has enabled us to identify some interactive strategies that the adult uses during triadic exchanges (Dimitrova and Moro, 2013; Dimitrova et al., 2015) as shown in Table 3. In the light of results, some strategies might be more effective in engaging infants in shared activities with objects (Trautman and Rollins, 2006; Serra et al., 2020). For example, Mendive et al. (2013) found that following and reinforcing an infant’s interest with an object (which they call *action maintenance*) favours the occurrence of coordinated episodes of triadic interaction. However, other strategies, such as proposing a new activity to the infant (*introduction*) or one different from the ongoing action (*redirection*), often result in passive observation or individual exploration of the object by the infant. This suggests that adult mediation should be adjusted to the infant’s developmental progress (De Schuymer et al., 2011; Zuccarini et al., 2018; Gattis et al., 2020), this also being key to observing the course of the action to provide responses that are consistent with the child’s actions and expressions of interest.

**Table 3:** Studies that reported adults’ verbal and/or behavioural strategies during triadic interactions (*n* = 13).

Reference	Topic	Infants’ age (in months)	Adults’ verbal strategies	Adults’ behavioural strategies
Danis et al. (2000)	Description of adults’ mediation in the emergence of prehension in infants.	2–4	Comments	Gestures
Moreno-Núñez et al. (2017)	Characterisation of adults’ communicative actions in home interactions.	2–4	Comments	Gestures and use of objects
Guevara et al. (2020)	Depiction of adults and infants use of gestures in nursery schools.	4–11	Verbal utterances (not specified)	Gestures and use of objects
Gattis et al. (2020)	Exploration of the emergence of attention sharing in infants.	5	Comments and directive speech	Gestures, use of objects and responsive actions
Legerstee et al. (2007)	Examination of affective attunement between adults and infants.	5–10	Comments, questions and responses to infants’ actions	Affective behaviour
Dimitrova and Moro (2013)	Illustration of how adults adjust to infants to construct shared knowledge.	8–16	N/A	Gestures
Dimitrova et al. (2015)	Characterisation of adults’ responses to infants’ gestures.	8–16	Questions and responses to infants’ actions	N/A
Dupertuis and Moro (2016)	Description of adults’ mediation in infants’ gesture development.	8–16	Comments	Use of objects
Mendive et al. (2013)	Identification of adults’ strategies to promote joint engagement with infants.	9	N/A	Responsive actions
Palacios et al. (2018)	Depiction of symbolic use of objects in infants.	9–15	N/A	Gestures and use of objects
Serra et al. (2020)	Identification of adults’ haptic patterns during play.	12	N/A	Affective behaviour
Trautman and Rollins (2006)	Exploration of the relationship between adults’ verbal utterances and infants’ communicative development.	12	Comments	N/A
Hobson et al. (2004)	Examination of adults’ sensitivity to infant’s engagement cues.	12–14	N/A	Responsive actions

These adjustments also appear to follow a dynamic development over time, which is reflected in different interaction behaviours. For example, Danis et al. (2000) observed variations in the content of maternal speech to the infant as a function of the infant’s progressively more sophisticated motor skills between the second and fourth month of life. While messages related to the physical properties of the object tended to decrease, in contrast, verbalisations referring to the infant’s hands, manipulative actions, and invitations to grasp the object increased. This is consistent with the findings of Dimitrova and Moro (2013), who observed how adults adapt their communicative action to the degree of knowledge they perceive in infants in relation to the use of the object: the more knowledge they attributed to the infant, the less gestural support they gave, and the less repetitive and exaggerated production of movements they made. In this way, the co-construction of communicative dynamics with these babies favoured development towards new learning.

While adult mediation is key to creating spaces for early interaction, the infants’ progressively more active participation favours the dynamic interactive patterns that both co-construct (Rollins and Greenwald, 2013; Schneider et al., 2022). Some studies suggest that, in situations of triadic interaction with objects, a symmetrical co-regulation prevails between both participants (Aureli et al., 2017). This is not observed in conditions of dyadic interaction (i.e., without objects), in which it is the adult who tends to attract, maintain, and redirect the infant’s attention. Likewise, certain routines such as shared reading activities appear to favour the active and dynamic involvement of both participants (Rossmanith et al., 2014).

Although it is true that both start from different levels of attention and coordination, the fluency and sophistication of their exchanges increases progressively over time. In this process, the episodes of individual exploration and social initiatives related to the first conventional uses of the object also tend to increase. This is further influenced by factors such as affective attunement (Legerstee et al., 2007; Rollins and Greenwald, 2013). This, combined with the fact that the infant is increasingly effective in combining a variety of communicative resources, seems to increase the likelihood of the adult’s response, who may more easily identify the child’s actions as “something to react to” (Moreno-Núñez et al., 2021b). Moreover, in recent years, several studies have been interested in how family socioeconomic status could be related to different conceptions and socialisation goals, which could influence the degree of initiative and interactional agency of infants (Abels and Hutman, 2015; Abels, 2020) and the development of triadic coordination skills (Gago-Galvagno and Elgier, 2020; Simaes et al., 2022).

#### 3.2.4. Triadic interactions as a shared learning framework

In addition to the analysis of how and when children’s first intentional behaviours emerge, the study of triadic interactions may help to understand other important developmental milestones and their social nature. For example, from the age of 6 months, the number of episodes of triadic interaction in which children engage is positively related to their anticipatory response to adult action (Brandone et al., 2019). This may be evidence of a progressive identification of others as intentional agents. In addition, recent research also proposes to approach the development of intentional understanding from enactive and embodied perspectives, which differ from the classical cognitivist paradigm: “It seeks to attend to the practical aspect of social cognition, that is, what subjects do when they intentionally understand others” (Vietri et al., 2021, p. 4).

According to these positions, infants’ own bodily activity and its congruence with adult action would constitute a basic form of intentional understanding. Infants’ anticipatory bodily adjustments during every-day routines, for example, when at mealtimes they lean forward and open their mouths to facilitate adults’ deliberate action with the spoon, appear to point to an early “understanding” of certain intentions. This lays the foundation for the origin of communicative intentionality in the child, from the first references shared with others. In this process, triadic interactions constitute a fundamental setting for the origin and development of various communicative mediators, such as gestures. In this sense, the pointing gesture has traditionally been considered one of the first communicative milestones. It allows Infants, towards the end of the first year, to interact with others and to communicate intentions, feelings, and requests about present but distant referents.

Notwithstanding, some studies included in this review question whether the establishment of shared reference is associated only with the pointing gesture, suggesting that ostensive gestures (e.g., giving and showing) also permit sharing interest in an object with the other, and could therefore contribute to the child’s understanding of the referent (Boundy et al., 2016; Guevara et al., 2020; Moreno-Núñez et al., 2020). In ostensive gestures, the referent occupies the hand itself and, therefore, these present less semiotic complexity than distal gestures. Children often address these gestures to themselves (Dupertuis and Moro, 2016), in a self-presentation of the object with exploratory and gradually reflexive functions. The transition between individual exploration of the object and the emergence of communicative behaviours directed to others would take place within the framework of triadic interactions. This enables the semiotic complexity of the interaction to be increased from the gradual distancing of the referent and the construction of socio-cultural meanings around the uses of objects (Moreno-Núñez et al., 2020).

Precisely, in the interaction with infants, adults rely on diverse repertoires of actions that include how they act with objects (Bialek et al., 2014). Thus, children receive a great deal of information about their environment, for example, mediated by the incorporation of musical elements and dynamics that organise the interaction and contribute to attracting the child’s attention and interest (Moreno-Núñez et al., 2015). This promotes the establishment of shared activity niches and favours affective and communicative harmony with the adult. These proposals have given rise to new methodological designs, which include the combination of software and analysis procedures that make it possible to detect patterns and variations in the musical resources present in the interactions (Alessandroni et al., 2020). Although adults readjust their mediation as the child gains in agency, they continue to support the acquisition of later skills such as evoking events or objects that are not present (Palacios and Rodríguez, 2015; Palacios et al., 2016, 2018) or language (West and Iverson, 2017). In relation to the latter, some studies have observed that adults’ verbal utterances during the child’s manipulative periods contain a higher proportion of labels, which facilitate object-word association and encourage interaction based on specific proposals for action (e.g., while playing with a phone and commenting: “Are you talking to Daddy? Say hello to Daddy”) (West and Iverson, 2017, p. 198).

## 4. Discussion and conclusions

The scientific literature on early social-communicative development has dealt extensively with the study of infants’ first triadic interactions with adults and objects, giving rise to different theoretical and methodological perspectives for their empirical study. The aim of this paper was to analyse some relevant issues on which there is still no consensus. To this end, recent scientific projects were explored, describing their methodological characteristics and discussing their results based on a thematic analysis. With respect to the distribution of the studies, we have been able to verify that most of the studies have been carried out in Western regions, mainly Europe and North America, thus reproducing a common bias in psychological research based on the study of WEIRD (Western, Educated, Industrialised, Rich, and Democratic) population samples. From a gender perspective, while this is not a study focused on gender disparities, it is striking that in most of the studies in our sample (except for those in which no details on gender were provided), adult data came from female participants, including mothers, educators, and researchers. Thus, to obtain a true picture of early socio-communicative development in everyday routines, it would be interesting for future research to explore the reasons behind this imbalance, as well as to promoting the participation of male adults in interaction studies with young children.

Moreover, the laboratory has been the predominant context for data collection, although there is a growing trend towards research in naturalistic contexts, such as the home. In this regard, technological advances in recent decades have led to the use of activity recording instruments that impact on the degree of ecological validity of the data. For example, some studies use head-mounted camera and eye-tracker systems to record infants’ field of vision and their visual trajectory during certain activities (Yu and Smith, 2013, 2016). However, we have not found studies that analyse triadic interactions by using *wearable* sensors, which could add accurate and relevant information about infants’ autonomy and/or motor activity when interacting in their environment (De Barbaro, 2019).

In relation to the methodological characteristics of the studies in our sample, there is a greater representation of descriptive studies as opposed to experimental designs, which are mainly longitudinal and cross-sectional. We consider this to be an important factor, especially at an early age, as longitudinal research allows for a better definition over time of the dynamic and changing processes related to the phenomena studied (Grimm et al., 2017). Finally, we have been able to observe how, in this sample, the use of quantitative strategies for data analysis prevails over qualitative or mixed methods approaches. Nonetheless, in qualitative studies, it should be noted that some of the techniques used could be particularly useful in the study of adult-infant interactions. For example, microgenetic procedures allow for in-depth and systematic analyses of the subtle changes that occur on a small scale in early development (Alessandroni et al., 2020; Lourenço et al., 2021). Furthermore, ethnographic studies such as that of Abels (2020), enrich observational data through researchers’ manual annotations during the continuous accompaniment of participants in their daily activities.

Subsequently, thematic analysis of the results has allowed us to identify different dilemmas about the nature of infants’ early experiences with adults and objects. First, research on joint attention and, specifically, on visual trajectories, has enabled us to approach the origins of adult-infant referential communication (Parise et al., 2007; Tremblay and Rovira, 2007; Perra and Gattis, 2012; Yu and Smith, 2013, 2016, 2017). Even when infants are limited by biological constrictions that prevent them from sustaining prolonged gaze, following the other’s gaze enables them to share references about the world (Flom et al., 2004; Cleveland and Striano, 2007; Osório et al., 2011).

Nonetheless, more recent work considers that joint attention goes beyond a simple coincidence of the gaze of two participants on the same object: during the interaction, they must also be attentive to the interlocutor’s behaviours and accommodate them, which implies understanding themselves and the other as intentional agents (Tomasello, 1995). For example, the adult takes advantage of the child’s bouts of attention to prolong exchanges by using various mediators, such as facial expression or language. This has led to an increasingly important role being given to exploration and communicative interaction, rethinking the study of phenomena associated with the beginnings of triadicity, based on the possibilities offered by materiality (Gibson, 1979). Accordingly, perception (attention) and action are part of the same process of active knowledge construction (Reddy, 2005; Heras Escribano, 2012). In this, attentional encounters would constitute the basis on which to establish instances of communication and joint action with others. These usually take place through objects and their cultural meanings (Moreno-Núñez et al., 2021b; Alessandroni, 2023), promoting learning that will gradually enable the child to operate coherently with the socio-material environment that surrounds them (Bruner, 1983; Rogoff, 1990; Carpenter et al., 1998).

This has led us to analyse other dilemmas surrounding the origin of the first triadic behaviours, such as when they emerge in the child’s development. In this regard, numerous studies agree that infants’ social-communicative skills become more sophisticated from the age of 9 months to a year (Trevarthen and Hubley, 1978; Tomasello et al., 2005), so a greater proportion of studies focussed on this period was expected (Flom et al., 2004; Parise et al., 2007; Osório et al., 2011; Mendive et al., 2013; Abels and Hutman, 2015; Abels, 2020). Despite this, it seems unlikely that infants’ communicative intentionality would suddenly emerge; on the contrary, it may emerge from early triadic encounters that adults organise around the object well before the age of 9 months (Striano and Bertin, 2005; Tremblay and Rovira, 2007).

Accordingly, the coordination of the processes necessary for triadic interaction would take place on the basis of the adult action (Amano et al., 2004; Cleveland and Striano, 2007) and infants’ early sensitivity to communicative cues, such as the exchange of glances at others and the object (Striano and Stahl, 2005). Consequently, before the infant can actively participate in triadic exchanges, the adult promotes their participation in a continuous process (Rodríguez and Moro, 1999) involving various semiotic mediators. On the one hand, they often resort to reiterative actions that favour the predictability of exchanges, making it easier for adults and infants to mutually “read” each other’s intentions over time (Alessandroni et al., 2020). On the other hand, adult mediation frequently relies on the use of objects and gives rise, as the infant acquires greater communicative tools, to actions which respond to the other’s behaviours and the cultural functionality of objects (Rossmanith et al., 2014; Moreno-Núñez et al., 2015, 2017; Cárdenas et al., 2020; Guevara et al., 2020).

Thus, aligned with a socio-constructivist and externalist approach to development, children build a shared “fund of knowledge,” which facilitates the learning of numerous skills (e.g., intentional understanding) and enables them to behave in a culturally situated manner (Barresi and Moore, 1996; Rochat, 2007; Brandone et al., 2019, 2020). Notwithstanding, these results must be considered in the light of some limitations. On the one hand, the underrepresentation of studies run in certain environments familiar to the child (e.g., nursery schools) and populations beyond the Western context, implies a bias that prevents extrapolation of some of the results. Future research aimed at a global understanding of early communicative processes (Amir and McAuliffe, 2020) should adopt an ecological perspective that responds to the everyday social, material, and cultural circumstances in which triadic interactions take place (Keller et al., 2009; Rowe and Goldin-Meadow, 2009; Abels, 2020).

Furthermore, the diversity of theoretical and conceptual interpretations of the origin and development of triadic interactions has made it necessary to adopt a qualitative approach in this review, aimed at describing and discussing the different positions. In contrast, a quantitative review (meta-analysis) would enable further exploration of some of the specific questions that arise from this study, such as the extent to which triadic interactions benefit early communicative development, or the relationship between different factors and children’s experiences of triadic interaction. For example, family socioeconomic status may be linked to different degrees of exposure to triadic interaction (Gago-Galvagno and Elgier, 2020), while atypical developmental trajectories (such as prematurity at birth, Down syndrome or high-risk for ASD) may result in difficulties over social participation (Chiang et al., 2008; Adamson et al., 2009; Benassi et al., 2016; Vilaseca et al., 2022) which can be improved through early intervention (Olafsen et al., 2006; Bejarano-Martín et al., 2022; Mattie and Fanta, 2023).

Further study of the development of early triadic interactions (i.e., from birth) could also provide possible improvements in early childhood education and care practises. For example, in the family context, some studies suggest that infants’ early communicative behaviours are not always effective in eliciting adult responses (Dimitrova et al., 2015; Boundy et al., 2016). Therefore, further exploration of infants’ cues in the context of early interactions around materiality could lead to the promotion of low-cost daily stimulation activities, such as shared reading practises (Black et al., 2017). This could also be relevant for exploring the impact of parental mental health in the development of early triadic interactions (e.g., in cases of postpartum depression or parental stress), as parental emotional regulation may affect their interactive and communicative styles (Apter and Devouche, 2019). In this sense, interventions aimed at accompanying parents by enhancing their interactive skills through video-feedback have been shown to boost caregivers’ self-perceived efficacy (Fisher et al., 2016) and contribute to improving parental mental health (Izett et al., 2021). In addition, they have also been shown to benefit infants’ development, for example, by fostering expressive and receptive communication skills (Imhof et al., 2023) or reducing behavioural problems (Liu et al., 2021).

Additionally, given the importance of the early years in psychological development and the importance of school as a socialising context, the findings synthesised in this review could be relevant for educators, as they could permeate educational processes to promote infants’ engagement with others and their environment. This requires further transferences between research and school, based on processes of joint reflection between professionals on their own educational practise. As is observed with parents at home, educators frequently mediate children’s participation in shared activities loaded with socio-cultural meanings and tools (Brossard, 2001; Rodríguez and De los Reyes, 2021). This takes place, again, through a multimodal prism in which objects, materials, language, gestures, and emotional expressions are usually involved (Estrada, 2021). Although these types of interactions are part of children’s everyday lives, it is surprising how little scientific literature to date has explored the educational practises at these early stages (0–3 years), particularly the role that material culture plays in shaping early development in various contexts.

The results of this review suggest that early triadic interactions: (1) are characterised as multimodal communicative exchanges that are structured in sequences of joint attention and action through materiality, (2) are built from the first months of life and develop thanks to adult mediation, and (3) constitute a privileged context for the construction of sociocultural meanings, which are fundamental for infants’ engagement with others and their environment.

## Author’s note

A Spanish-language version of this article can be found online in the Supplementary material.

## Data availability statement

Publicly available datasets were analysed in this study. This data can be found here: https://doi.org/10.5281/zenodo.8134795.

## Author contributions

AM-G: conceptualization, methodology, data curation, analysis, investigation, and writing – original draft. AM-N: conceptualization, writing – original draft, writing – review & editing, and supervision. All authors contributed to the article and approved the submitted version.

## Funding

This study was supported by the Predoctoral Contract Scholarship for PhD Training (call 2020), granted to AM-G [reference PRE2020-094773], and project PID2019-108845GA-I00/AEI/10.13039/501100011033, both co-funded by the State Research Agency (Ministry of Science and Innovation, Spain) and the European Social Fund (ESF).

## Conflict of interest

The authors declare that the research was conducted in the absence of any commercial or financial relationships that could be construed as a potential conflict of interest.

## Publisher’s note

All claims expressed in this article are solely those of the authors and do not necessarily represent those of their affiliated organizations, or those of the publisher, the editors and the reviewers. Any product that may be evaluated in this article, or claim that may be made by its manufacturer, is not guaranteed or endorsed by the publisher.
