# Supplementary material for: Early triadic interactions in the first year of life: a systematic review on object-mediated shared encounters
Source: Front Psychol. 2023 Aug 17;14:1205973. doi: 10.3389/fpsyg.2023.1205973 (PMC10478714; doi:10.3389/fpsyg.2023.1205973)
Supplement: Supplementary file 1 [file Data_Sheet_1.pdf]

Supplementary Material

Las interacciones triádicas tempranas en el primer año de vida: una revisión sistemática sobre los encuentros compartidos mediados por objetos

Ana Mendoza-García[1], Ana Moreno-Núñez[1*]

[1]Departamento de Psicología Evolutiva y de la Educación, Universidad Autónoma de Madrid, Madrid, España

*** Correspondencia:** Ana Moreno-Núñez: ana.moreno@uam.es

Palabras clave: interacciones triádicas, comunicación referencial, acción conjunta, materialidad, desarrollo temprano.

Resumen

Las interacciones tempranas de los bebés con los adultos y objetos cotidianos son clave para el desarrollo socio-comunicativo, pero su aparición y desarrollo siguen siendo objeto de debate. Con el objetivo de describir los distintos enfoques teóricos y metodológicos sobre la triadicidad durante el primer año de vida, hemos realizado una revisión sistemática y cualitativa de la literatura reciente. Siguiendo las directrices PRISMA 2020, hemos explorado la producción científica de las últimas décadas acerca de las interacciones triádicas hasta los 12 meses de edad. Inicialmente, revisamos y filtramos 1943 artículos de los que obtuvimos una muestra final de 51 publicaciones. Los estudios se desarrollan normalmente en entornos de laboratorio, aunque la investigación ecológica es cada vez más común, especialmente en el hogar. A través del análisis temático de los datos, discutimos las diferentes perspectivas sobre el origen y la conceptualización de las interacciones triádicas, y cómo contribuyen a estructurar y facilitar otros fenómenos del desarrollo, como los gestos comunicativos y usos de los objetos. Antes de la aparición de la comunicación intencional, los adultos facilitan formas tempranas de triadicidad con los niños basadas en la creación de oportunidades de comunicación e interacción, tanto con los adultos como con la materialidad. Sin embargo, es necesario seguir investigando el potencial de las interacciones triádicas tempranas en el marco de las prácticas de crianza y acciones pedagógicas en educación infantil.

1 Introducción

Las primeras experiencias sociales de los bebés tienen lugar, desde los primeros meses de vida, a través de las interacciones con sus cuidadores y con objetos cotidianos (Moreno-Núñez, Fernández-Alcaide y Martín-Ruiz, 2021). Esto es coherente con el enfoque cultural-histórico y, más concretamente, con los postulados de la teoría de la actividad desarrollados a partir de las propuestas vygotskianas, desde las que se defiende que los individuos actuamos de manera colectiva en base a actividades compartidas e intercambios comunicativos que nos permiten apropiarnos de herramientas culturales (Foot, 2014; Vygotsky, 1978). Según esto, las interacciones sociales, especialmente con los adultos, resultan esenciales durante la primera infancia en tanto favorecen un espacio inter-comunicativo de mediación

y colaboración que facilita el aprendizaje y desarrollo del niño (Veraksa y Veraksa, 2018; Vygotsky, 1984).

La mediación del adulto hace referencia al proceso guiado para dotar a los niños, mediante actividades compartidas, de las herramientas psicológicas que necesitan para desenvolverse en un contexto sociocultural específico. Esto les permite internalizar progresivamente sus aprendizajes y, consecuentemente, desenvolverse en el mundo de forma cada vez más autónoma (Vygotsky, 1934/1986). A lo largo del primer año, la relación de los bebés con su entorno se va modificando a partir del acompañamiento del adulto (McCune y Zlatev, 2015; Vygotsky, 1932/1996), quien responde a sus primeras señales e intentos comunicativos a la vez que comparte la atención y actúa sobre objetos o eventos concretos (Bakeman y Adamson, 1984; Solovieva y Quintanar, 2021). Este proceso no se da, por tanto, en el vacío, sino en el marco de interacciones tempranas que tienen lugar en un contexto socio-material determinado. En ellas, los objetos se incorporan no como meras realidades materiales, sino como vehículo comunicativo entre bebé y adulto, incentivando la acción conjunta a partir de sus propiedades sociales y culturales (Burner y Svendsen, 2020; Rodríguez, 2012). Los significados y usos convencionales de los objetos se transmiten, así, en la interacción (Rodríguez, 2006; Solovieva et al., 2020) permitiendo al bebé sofisticar sus acciones y convertirlas progresivamente en actos sociales dirigidos al otro y coordinados conjuntamente (Carpendale et al., 2021).

El papel de los encuentros triádicos (adulto-bebé-objeto) en el desarrollo socio-comunicativo ha sido el núcleo de un amplio debate, especialmente con relación a su origen. Tradicionalmente, se ha considerado que durante los primeros meses de vida los bebés se relacionan primero con los adultos y, algo más tarde, también con los objetos. Ello ha dado lugar a la diferenciación entre intersubjetividad primaria, referida a los intercambios diádicos tempranos entre adultos y bebés, e intersubjetividad secundaria, en las que las interacciones pasarían a incorporar referentes externos, adquiriendo un carácter triádico (Carpenter et al., 1998; Striano y Reid, 2006; Tomasello et al., 2005; Trevarthen y Hubley, 1978). El paso de una etapa a otra suele situarse en torno a los nueve meses de edad, cuando emergen los primeros actos comunicativos intencionales del bebé hacia el adulto en lo que Tomasello (2013) denomina la "*revolución de los nueve meses*". A partir de entonces, aumenta la complejidad de las conductas socio-comunicativas del niño, ya que es capaz de compartir experiencias con los demás mediante, por ejemplo, el uso de gestos y la diversificación de funciones comunicativas (Bates et al., 1975).

Sin embargo, trabajos más recientes cuestionan que la aparición de las primeras experiencias triádicas de los bebés se limite al final del primer año, caracterizándolos como un proceso continuo y gradual mediado por el adulto desde el nacimiento (Reddy, 2010; Rodríguez, 2006). Desde este punto de vista, los inicios de la comunicación se desarrollarían desde el inicio de la vida de acuerdo a procesos de interacción dinámicos (Fogel, 1993; Fogel y Thelen, 1987; Thelen y Smith, 1994) en el que lo que se comparte no se transmite unidireccionalmente, de una mente a otra, sino que se crea conjuntamente entre adulto y niño (Fogel, 1993; Trevarthen y Reddy, 2017) o en el marco de dinámicas familiares con más de un cuidador principal (Fivaz-Depeursinge y Corboz-Warnery, 1999; León y Olhaberry, 2020). La intencionalidad comunicativa, por tanto, se co-construye e integra gradualmente como parte del repertorio de habilidades del bebé, a partir de los encuentros que media el adulto a lo largo del tiempo (Rączaszek-Leonardi et al., 2019) y en los que con frecuencia incorporan objetos para comunicarse sobre -y a través- del mundo material (Rodríguez, 2012).

Precisamente, la falta de atención prestada a la mediación a través de objetos en las interacciones tempranas es otra de las problemáticas que subyacen a su conceptualización. Desde posiciones clásicas,

las interacciones triádicas con objetos se basan en episodios de *atención conjunta*, que pueden entenderse desde diferentes perspectivas (Gabouer y Bortfeld, 2021). Por un lado, desde una perspectiva asociativa dependerían del sistema de orientación visual del bebé y su capacidad para seguir la mirada del otro (Butterworth, 1991), lo que se considera que ocurre entre los seis y los 18 meses de edad en ausencia de procesos interactivos (Butterworth y Jarrett, 1991; Corkum y Moore, 1998). Por otro lado, desde una perspectiva social se destaca la coincidencia de miradas entre adulto y bebé sobre un referente, en el marco de un proceso de interacción dinámico entre el niño y su entorno que comienza al inicio del primer año de vida (Striano y Stahl, 2005) pero continúa desarrollándose a lo largo de todo el segundo año (Tomasello y Farrar, 1986). Algunos trabajos enfatizan estos procesos de co-regulación atencional, comunicativa y afectiva entre adultos y bebés sobre un objeto de interés mutuo que se desarrolla entre los seis meses y el año y medio de edad. De este modo, distinguen entre "engagement coordinado", que alude a los componentes multimodales que se dan en los episodios intersubjetivos (e.g., sonrisas o gestos), y "engagement pasivo", que se limitaría a la coincidencia de focos atencionales sobre un mismo objeto (Bakeman y Adamson, 1984).

Si bien el paradigma de la atención conjunta ha ocupado un papel central en la investigación de las últimas décadas, estudios recientes invitan a modificar el prisma analítico a partir del creciente interés por los componentes multimodales en los que se apoyan estos encuentros entre el adulto y el niño (Yu y Smith, 2016, 2017). Por ejemplo, los adultos tienden a acompañar los episodios de atención conjunta combinando el input verbal y la estimulación táctil hacia el bebé (Suárez-Rivera et al., 2019), lo que podría estar asociado con una mayor duración de la mirada del niño hacia el objeto en comparación con los episodios no multimodales. Esto ha hecho cuestionar el estudio de la atención conjunta como un proceso aislado, ya que podría formar parte de un conglomerado de fenómenos cognitivos, sociales y comunicativos (Jacobson y Degotardi, 2022; Siposova y Carpenter, 2019) que tienen lugar en dinámicas de *acción conjunta* entre el bebé y los otros (Reddy, 2005). Adoptar una perspectiva interactiva y multimodal permitiría enfatizar no sólo los aspectos atencionales, sino también otros elementos de la interacción como la emoción, el tacto o los usos de los objetos (De Barbaro et al., 2013; Moreno-Núñez et al., 2017; Rossmanith et al., 2014).

La falta de un consenso establecido en torno a las interacciones triádicas, por ejemplo, acerca de cómo y cuándo surgen o sobre las características particulares que se derivan de su conceptualización, ha abierto un debate teórico y metodológico. Por tanto, existen motivos para identificar esas áreas inconclusas y explorar las diferentes perspectivas sobre el origen, el desarrollo y la naturaleza de las interacciones triádicas. Para ello, realizamos una revisión sistemática y cualitativa dirigida a explorar la producción científica de las últimas décadas y sus principales hallazgos, discutiendo sus implicaciones potenciales para el estudio del desarrollo temprano y la atención a la primera infancia. De acuerdo a esto, nuestro trabajo podría ser informativo para la futura exploración empírica de este fenómeno.

2 Método

Siguiendo las recomendaciones de la declaración PRISMA 2020 (Page et al., 2021), una versión actualizada de PRISMA 2009 (Moher et al., 2009), realizamos una búsqueda bibliográfica exhaustiva en ocho bases de datos: Web of Science, Scopus, ProQuest, PubMed, ScienceDirect, PsycINFO, Dialnet, y Dimensions. Para ello, partimos de cuatro palabras clave, en inglés y en español, que se combinaron con operadores booleanos de acuerdo con la siguiente ecuación de búsqueda: ("interacciones triádicas") AND objetos AND infancia. Además de los artículos resultantes de esta búsqueda inicial, se incluyeron en el análisis otros estudios relevantes recuperados a partir de la bibliografía citada en estas investigaciones.

El diagrama de flujo PRISMA (Figura 1) ilustra el proceso de selección de los estudios para esta revisión. El número total de resultados obtenidos en la búsqueda inicial fue de 1943 artículos, de los que se eliminaron 297 duplicados. A continuación, se recogió la información general de cada estudio (autor, año de publicación, título, base de datos de procedencia y resumen) y se revisó su adecuación a la temática de estudio a partir del título y resumen. Esto supuso la exclusión de 1356 artículos que no guardaban relación con nuestros objetivos, ya que se centraban, por ejemplo, en salud mental parental, intervenciones en el marco familiar o investigaciones con primates no humanos.

A partir de la base de datos resultante (n= 290), se analizaron los textos completos y se cribaron de acuerdo con los siguientes criterios de elegibilidad:

- *Tipo de estudio*: se seleccionaron solo artículos empíricos publicados en revistas científicas revisadas por pares. Se excluyeron las tesis doctorales, capítulos de libros, monográficos, artículos teóricos y revisiones.

- *Área de estudio:* se incluyeron estudios que aportaran resultados en términos psicológicos (i.e., desarrollo humano o conducta).

- *Objetivo del estudio:* se seleccionaron publicaciones que exploraran las interacciones triádicas entre adultos, objetos y bebés. Los artículos sobre temáticas relacionadas (e.g., atención conjunta) se incluyeron solo si hacían referencia a la triadicidad de las interacciones estudiadas.

- *Edad de los participantes:* dado nuestro interés en analizar las interacciones tempranas, la revisión parte de las investigaciones centradas en el primer año de vida. No obstante, también se han tenido en cuenta aquellos estudios longitudinales en los que la recogida de datos se extendía hasta, como máximo, los 18 meses de los participantes.

- *Idioma:* se han incluido artículos publicados en inglés o español.

- *Fecha de publicación:* con el objetivo de realizar un análisis actualizado, solo se han considerado los trabajos publicados entre 2000 y 2022.

- *Acceso a texto completo:* se excluyeron de la muestra final aquellos artículos a los que no se haya podido acceder al texto completo para su revisión.

Las discrepancias sobre la inclusión de determinados artículos se resolvieron mediante consenso entre las investigadoras. El proceso de cribado dio lugar a una muestra final de 51 publicaciones, las cuales fueron sometidas a un análisis de calidad metodológica mediante QualSyst (Kmet et al., 2004). Esta herramienta proporciona listas de verificación adaptadas a un amplio rango de metodologías y temáticas que permiten la evaluación crítica de trabajos científicos cualitativos y cuantitativos. En base a ello, ofrece una estimación de la calidad metodológica de los estudios como resultado de una puntuación entre 0 y 1, siendo 1 la mayor calidad posible. Asimismo, propone distintos puntos de corte para decidir sobre la exclusión de los trabajos, que varían entre 0.55 y 0.75. Todos los estudios incluidos en nuestra muestra final superan el punto de corte más alto, 0.75 (M= 0.925; SD= 0.06).

Figura 1. Diagrama de flujo PRISMA

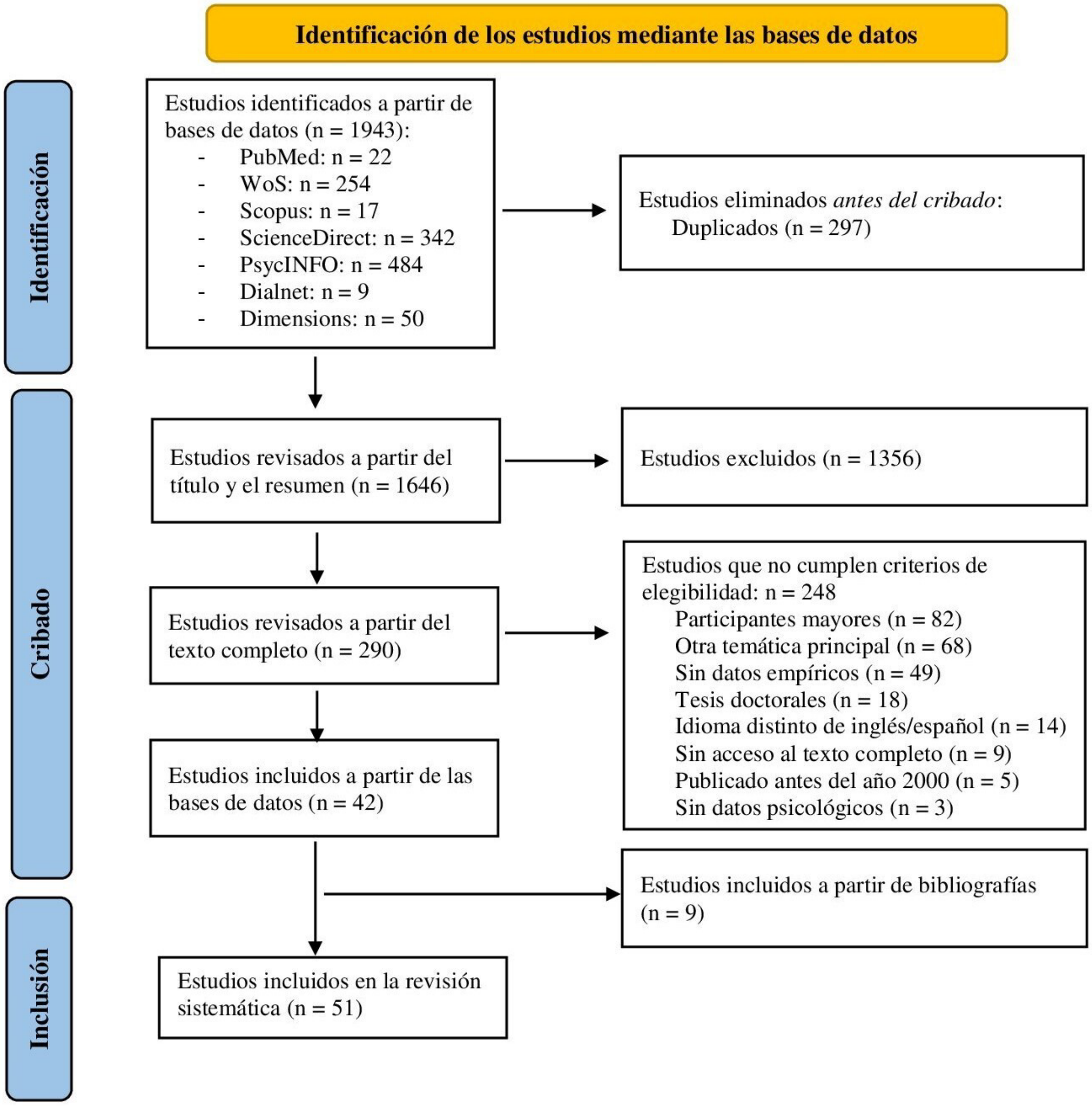

3 Resultados

Para el análisis, en primer lugar, exploramos la distribución de la producción científica incluida en la muestra con respecto a sus características poblacionales (lugar de realización de los estudios y contexto en el que se recogieron los datos) y metodológicas (tipo de estudio, tipo de diseño, tipo de tarea de interacción y estrategia de análisis de datos). En segundo lugar, realizamos un análisis temático de los resultados a partir de la discusión sobre el origen, desarrollo e implicaciones educativas de las interacciones triádicas.

3.1 Distribución de la producción científica reciente

Al analizar los datos sobre el lugar de realización de los estudios (Figura 2) observamos que, entre las publicaciones analizadas, predominan los estudios desarrollados en países europeos (59%) y Norteamérica (25%). No obstante, probablemente debido a que nuestra revisión incluye también textos en español, la muestra comprende un considerable porcentaje de estudios desarrollados en países

latinoamericanos (12%). Llama la atención la escasa representación de investigaciones realizadas en contextos asiáticos (4%), con sólo dos estudios desarrollados en Japón e India. No hemos encontrado ningún trabajo en esta temática a partir de datos recogidos en Oceanía.

Figura 2. Distribución de la muestra respecto al lugar de realización de los estudios

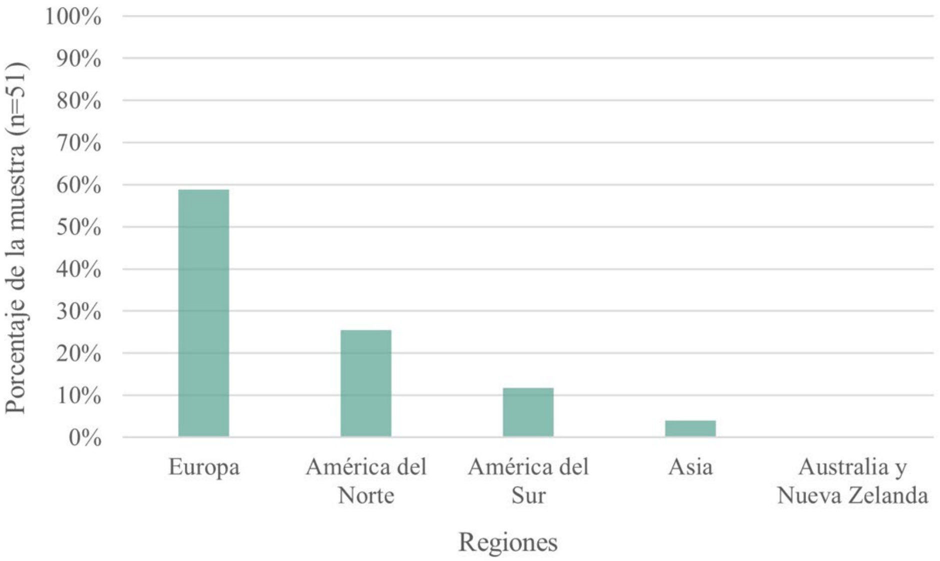

Asimismo, consideramos importante explorar las tendencias que, en los últimos años, ha seguido el estudio de las interacciones triádicas en el marco de diferentes contextos de la vida cotidiana del niño (Figura 3), ya que estos podrían dar lugar a diferentes formas de interactuar con otros y con objetos del entorno. Con este objetivo, definimos cuatro categorías que clasifican las interacciones analizadas en función del grado de control de variables: laboratorio, hogar, escuela infantil y mixtos (i.e., estudios que combinaban dos o más de las anteriores). El laboratorio ha sido y es el contexto predominante en la recogida de datos de la mayoría de las investigaciones analizadas (53%). No obstante, se observa un aumento reciente de estudios que parten de datos obtenidos en contextos naturales, especialmente el hogar (41%). Ello incluye estudios en los que las interacciones se dan entre el niño y su madre/padre en un entorno familiar para el bebé (por ejemplo, su propia casa o su escuela). Sin embargo, otras interacciones igualmente cotidianas en la vida del niño, como las que se desarrollan con sus educadoras e iguales en la escuela infantil, se encuentran aún poco representados en la literatura (4%). Por último, a pesar de que solo hemos encontrado un estudio de estas características (2% de la muestra), nos parece importante destacar que podríamos encontrar estudios mixtos que combinan datos obtenidos en más de uno de estos contextos, por ejemplo, el hogar y el laboratorio (Danis et al., 2000). Sin embargo, esta no es una alternativa que se haya mantenido en el tiempo, a favor de estudios centrados en uno u otro contexto.

Figura 3. Distribución de la muestra por año de publicación respecto al contexto en el que se desarrollaron las interacciones

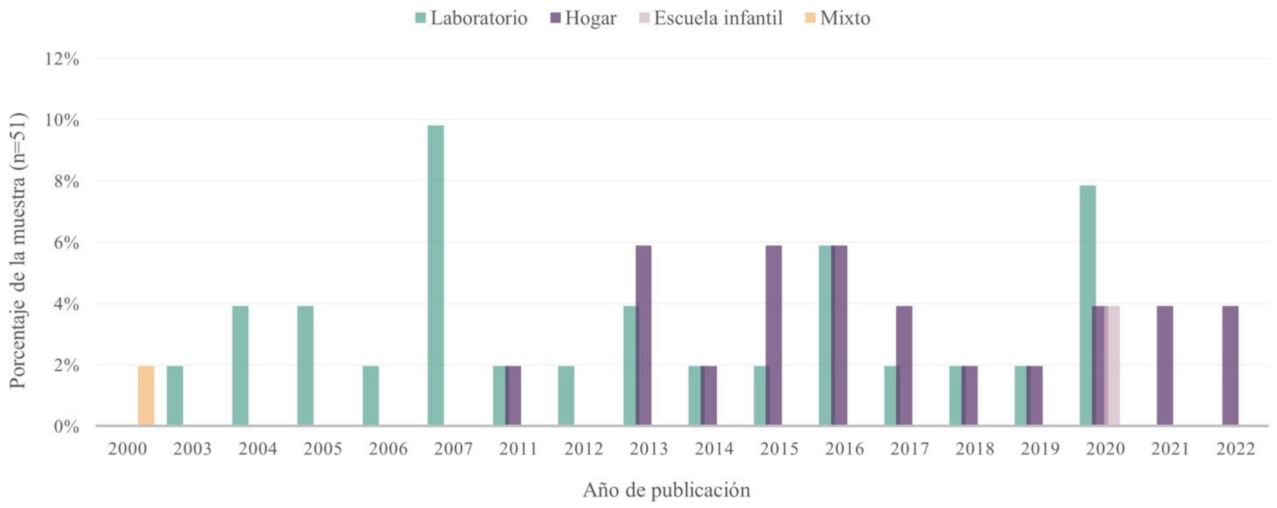

Por último, la Tabla 1 recoge la distribución de la muestra en cuanto a tres elementos: primero, respecto al tipo de estudio; segundo, en relación con el tipo de diseño empírico y tercero, a partir de la estrategia seguida para el análisis de datos (Montero y León, 2002). Para mayor exhaustividad, se incluye también el tipo de tarea de interacción que utilizaron en los diferentes estudios de nuestra muestra. Respecto al tipo de estudio la muestra comprende eminentemente investigaciones de carácter descriptivo (71%) y experimental (29%). En relación con el tipo de diseño empírico, observamos una distribución más homogénea entre diseños longitudinales (55%) y transversales (43%), mientras que los diseños pre-post test en esta área son escasos (2%). Los estudios se basaron mayoritariamente en interacciones de juego libre entre adultos (tanto investigadores como padres) y bebés (74,5%), seguidas de interacciones experimentales (17,5%) dirigidas a elicitar determinados comportamientos en los niños. Se encontraron muy pocos estudios basados en rutinas cotidianas en el hogar, como la alimentación (4%) o en interacciones guiadas por educadoras en escuelas infantiles (4%). Aunque no todos los estudios proporcionaron datos sobre el tipo de objetos utilizados, la gran mayoría usaron juguetes (incluidos los juguetes musicales y cuentos) y artefactos (por ejemplo, una cuchara) para promover la interacción. Por último, de acuerdo con la estrategia de análisis de datos, existe una predominancia significativa de estudios cuantitativos (78%) en comparación con las investigaciones cualitativas (10%) o que emplean métodos mixtos (12%).

Tabla 1. Frecuencia y porcentaje de estudios en la muestra (n=51) según el tipo de estudio, el tipo de diseño, el tipo de tarea de interacción y la estrategia de análisis de datos

		No. de estudios	% de la muestra
Tipo de estudio	Descriptivo	36	71%
	Experimental	15	29%
Tipo de diseño	Longitudinal	28	55%
	Transversal	22	43%
	Pre-post test	1	2%
Tipo de tarea	Interacciones de juego libre	38	74,5%
	Interacciones experimentales	9	17,5%
	Interacciones guiadas por la educadora	2	4%
	Rutinas cotidianas	2	4%

	Cuantitativo	40	78%
Estrategia de análisis de datos	Cualitativo	5	10%
	Mixto	6	12%

3.2 Análisis temático de los datos

La segunda parte de este estudio consistió en un análisis temático de los datos (Braun y Clarke, 2006), para lo que organizamos los resultados en base a cuatro ejes de discusión: (1) los fenómenos asociados a los inicios de la triadicidad, (2) el origen de las primeras conductas triádicas, (3) el proceso de co-construcción de la interacción entre adulto y bebé, y (4) la función de las interacciones triádicas como marco de aprendizaje compartido.

3.2.1 Los primeros encuentros triádicos: de la atención a la acción conjunta

Uno de los fenómenos más estudiados sobre el origen de la triadicidad en la primera infancia es la atención conjunta, un proceso inherentemente socio-comunicativo, basado en la cognición social y la intencionalidad comunicativa. Si bien numerosas investigaciones han circunscrito su estudio empírico a aspectos relacionados con el sistema de orientación visual del bebé, por ejemplo, mediante medidas como la fijación de la mirada y/o el seguimiento de trayectorias visuales entre los tres y los 12 meses de edad (Cleveland y Striano, 2007; Gattis et al., 2020; Parise et al., 2007; Tremblay y Rovira, 2007), otros trabajos proponen que la atención conjunta debería describirse a partir de las habilidades socio-comunicativas del bebé y del adulto, que median en las interacciones con objetos a lo largo de todo el primer año de vida (Osório et al., 2011; Striano et al., 2007; Striano y Bertin, 2005; Striano y Stahl, 2005). Entre ellas, el seguimiento de la mirada del otro parece erigirse como un componente esencial desde los primeros meses de vida (Perra y Gattis, 2012) aunque no es el único.

Existen más conductas que permiten sostener la interacción y participar de ella, por ejemplo, el seguimiento visual de los gestos indiciales del adulto que emerge sobre los tres o cuatro meses de edad y las manipulaciones del objeto en el último tercio del primer año (Amano et al., 2004; Flom et al., 2003). Aunque estos trabajos han realizado importantes contribuciones al estudio del desarrollo perceptivo-atencional temprano, su punto de vista resulta limitado en tanto no incorpora al análisis otras variables propias de las relaciones psicológicas entre adultos, objetos y bebés, como son sus características multimodales o la construcción de significados compartidos en la interacción.

Esta problemática, en parte, deriva de la falta de atención desde este enfoque hacia el papel que desempeñan los referentes en las interacciones triádicas. Los objetos y sus normas culturales de uso favorecen y catalizan las interacciones al constituirse como un referente compartido, por lo que resultan clave al analizar los intercambios triádicos, especialmente a edades tempranas (Alessandroni et al., 2020; Dimitrova y Moro, 2013; Dupertuis y Moro, 2016; Moreno-Núñez et al., 2017; Palacios y Rodríguez, 2015; Vietri et al., 2021). En un estudio a partir de situaciones de lectura compartida, Rossmanith et al. (2014) describen cómo los adultos, desde los tres meses de edad del bebé, convierten el objeto en un elemento tanto al que atender (por ejemplo, mostrando el libro y empleando sonidos, vocalizaciones y verbalizaciones), como también sobre el que actuar juntos (pasando páginas, abriendo solapas desplegables, tocando distintas texturas o señalando los dibujos). Desde este punto de vista, la atención y la acción conjunta son dos partes indisociables de intercambios socio-comunicativos, multimodales y complejos, en los que los objetos ejercen como vehículo comunicativo entre adultos y

bebés (Moreno-Núñez, Murillo et al., 2021). Al inicio, son los adultos quienes dotan estos encuentros de un carácter triádico, integrando secuencias de atención y acción conjunta y facilitando progresivamente el aprendizaje de los bebés sobre cómo relacionarse y operar en el mundo.

3.2.2 Los orígenes del desarrollo de la triadicidad

Dadas las distintas perspectivas acerca de cuándo emergen las primeras interacciones triádicas, nos planteamos si esto pudiera verse reflejado en los diseños representados en la muestra. Por ejemplo, concebir la triadicidad como una habilidad que emerge sólo a partir de los nueve meses podría tener un impacto en la selección de la edad de los participantes, y dar lugar a un menor volumen de evidencias respecto a lo que ocurre en meses anteriores. Para ello, exploramos la distribución de los estudios en función de las edades analizadas (en meses). Puesto que algunos estudios abordan un rango de edad determinado, cada edad se ha considerado como categoría no mutuamente excluyente. Así, en la Tabla 2 se observa que existe una mayor proporción de estudios en la muestra cuanto mayor es la edad de los bebés, especialmente durante el último tercio del primer año de vida, donde se concentra el 53% del total de observaciones (Abels, 2020; Abels y Hutman, 2015; Flom et al., 2004). Estos estudios aportan información relevante sobre los procesos de atención y acción conjunta a partir del momento en el que los bebés ya poseen cierta destreza en la comunicación triádica (Mendive et al., 2013; Osório et al., 2011; Parise et al., 2007) pero, al poner el foco únicamente en cuándo los niños adquieren el logro, dejan por resolver cómo se fundamentan estas habilidades en etapas más tempranas.

Tabla 2. Frecuencia y porcentaje de estudios en la muestra (n=51) según la edad en meses de los participantes

	**Edad (meses)**												
	1	2	3	4	5	6	7	8	9	10	11	12	> 12
No. de estudios	1	6	12	17	15	18	16	19	29	25	24	29	15
% de la muestra	2%	12%	24%	33%	29%	35%	31%	37%	57%	49%	47%	57%	29%

A pesar de lo anterior, en las últimas décadas se han incrementado los estudios que, de forma específica, atienden a las competencias socio-comunicativas tempranas (Amano et al., 2004; Striano y Bertin, 2005; Striano et al., 2007; Tremblay y Rovira, 2007). Por ejemplo, Striano y Stahl (2005) ya sugerían que las habilidades triádicas se desarrollan progresivamente a lo largo de los primeros meses de vida, a partir de un estudio comparativo en función de la duración de la mirada de los bebés. Para ello, diseñaron dos condiciones experimentales: en la primera, el adulto presentaba un objeto al bebé acompañándose de comentarios en tono afectuoso, mientras coordinaba la atención visual entre el objeto y el niño; en la segunda, el adulto presentaba de igual forma el objeto, pero esta vez sólo dirigiendo su mirada hacia este. Encontraron que, ya a los tres meses, la duración de la mirada de los bebés era superior en la primera condición experimental, lo cual podría evidenciar cierta sensibilidad temprana hacia señales comunicativas que son relevantes en la interacción triádica, como el contacto visual con el adulto en relación con el objeto.

Ello destaca el papel que desempeña el adulto en los intercambios y rutinas cotidianos del bebé con su entorno y, concretamente, con el mundo material. Recientemente, más trabajos apuntan a que el adulto involucra activamente al bebé y al objeto en un mismo acto comunicativo desde los primeros meses de vida (De Barbaro et al., 2013, 2016; Moreno-Núñez et al., 2017; Rossmanith et al., 2014), generando interacciones triádicas *tempranas* aun cuando el niño no es todavía capaz iniciarlas por sí mismo

(Moreno-Núñez et al., 2017). Desde el nacimiento, adultos y bebés participan conjunta y habitualmente en acciones iterativas con objetos, que dotan de un carácter organizado a la interacción (Alessandroni et al., 2020; Moreno-Núñez et al., 2015). El entorno reiterativo y estructurado en el que se desarrollan las primeras interacciones triádicas favorece, además, la sintonía y comprensión de las acciones entre bebés y adultos (Dimitrova et al., 2015). Las redundancias creadas, por ejemplo, a través de la estimulación mediante el tacto, podrían darse en respuesta a aquellas situaciones en las que la mediación afectiva del adulto se hace más necesaria, bien porque se trata de interacciones con bebés muy pequeños (Alessandroni et al., 2020) o para acompañar al niño en sus intentos de completar una tarea difícil (Serra et al., 2020).

3.2.3 Co-construyendo la interacción: el papel del adulto en la progresiva agencialidad del bebé

En línea con esta idea de interacciones triádicas tempranas, es decir, antes de los nueve meses, cada vez más investigaciones buscan describir el papel del adulto en el establecimiento de las primeras referencias compartidas con el niño (Danis et al., 2000; Hobson et al., 2004; Moreno-Núñez et al., 2017). Ello ha permitido identificar qué estrategias interactivas usa el adulto durante los intercambios triádicos (Dimitrova et al., 2015; Dimitrova y Moro, 2013), como muestra la Tabla 3. A la luz de estos resultados, algunas estrategias podrían resultar más efectivas para involucrar a los niños en actividades compartidas con objetos (Serra et al., 2020; Trautman y Rollins, 2006). Por ejemplo, Mendive et al. (2013) observaron que seguir y reforzar el interés del bebé con un objeto (a lo que denominan *mantenimiento* de la acción) favorece la aparición de episodios de interacción triádica coordinada. Sin embargo, otras estrategias como proponer al bebé una actividad nueva (*introducción*) o diferente a la acción en curso (*redirección*), suelen dar lugar a la observación pasiva o la exploración individual del objeto por parte del niño. Ello sugiere que la forma en la que los adultos median en la interacción debe ajustarse al desarrollo evolutivo del bebé (De Schuymer et al., 2011; Gattis et al., 2020; Zuccarini et al., 2018), siendo igualmente clave observar el curso de la acción para dar respuestas coherentes con las acciones y muestras de interés del niño.

Tabla 3. Estudios que informan sobre las estrategias verbales y/o conductuales de los adultos durante las interacciones triádicas (n=13)

Referencia	Tema de estudio	Edad de los niños (meses)	Estrategias verbales del adulto	Estrategias conductuales del adulto
Danis et al., 2000	Descripción de la mediación de los adultos en la aparición de la prensión en los bebés	2-4	Comentarios	Gestos
Moreno-Núñez et al., 2017	Caracterización de las acciones comunicativas de los adultos en las interacciones en el hogar	2-4	Comentarios	Gestos y uso de objetos
Guevara et al., 2020	Descripción del uso de gestos de adultos y bebés en escuelas infantiles	4-11	Emisiones verbales (sin especificar)	Gestos y uso de objetos
Gattis et al., 2020	Exploración de la aparición de la atención compartida en los bebés	5	Comentarios y habla directiva	Gestos, uso de objetos y acciones responsivas
Legerstee et al., 2007	Exploración de la sintonía afectiva entre adultos y bebés	5-10	Comentarios, preguntas y respuestas a la acción de los niños	Conducta afectiva

Dimitrova y Moro, 2013	Ilustración de cómo los adultos se ajustan a los bebés para construir un fondo de conocimiento compartido	8-16	N/A	Gestos
Dimitrova et al., 2015	Caracterización de las respuestas de los adultos a los gestos de los bebés	8-16	Preguntas y respuestas a la acción de los niños	N/A
Dupertuis y Moro, 2016	Descripción de la mediación de los adultos en el desarrollo de los gestos infantiles	8-16	Comentarios	Uso de objetos
Mendive et al., 2013	Identificación de las estrategias del adulto que promueven la participación conjunta con los bebés	9	N/A	Acciones responsivas
Palacios et al., 2018	Descripción de los usos simbólicos de los objetos en bebés	9-15	N/A	Gestos y uso de objetos
Serra et al., 2020	Identificación de los patrones hápticos de los adultos durante el juego	12	N/A	Conducta afectiva
Trautman y Rollins, 2006	Exploración de la relación entre las emisiones verbales del adulto y el desarrollo comunicativo de los bebés	12	Comentarios	N/A
Hobson et al., 2004	Exploración de la sensibilidad de los adultos en relación con las señales comunicativas de los bebés	12-14	N/A	Acciones responsivas

Los ajustes del adulto parecen seguir, también, un desarrollo dinámico a lo largo del tiempo, que se ve reflejado en diferentes comportamientos propios de la interacción. Por ejemplo, Danis et al. (2000) observaron variaciones en el contenido del habla materna dirigida al niño en función de la progresiva sofisticación de las habilidades motrices del bebé entre el segundo y el cuarto mes de vida. Mientras que los mensajes relacionados con las propiedades físicas del objeto tendían a disminuir, aumentaban las verbalizaciones referidas a las manos, las acciones manipulativas del bebé y las invitaciones a agarrar el objeto. Esto es coherente con los hallazgos de Dimitrova y Moro (2013), que observaron cómo los adultos adaptan su acción comunicativa al grado de conocimiento de los objetos que perciben en los bebés: si creían que los bebés conocían mejor el objeto, reducían sus apoyos gestuales, realizaban menos repeticiones y disminuían la exageración en algunos movimientos. De este modo, la co-construcción de dinámicas comunicativas con los bebés favorece el desarrollo hacia nuevos aprendizajes.

Si bien el rol mediador del adulto es clave para generar espacios de interacción temprana, la participación progresivamente más activa de los bebés favorece los patrones dinámicos de interacción que ambos co-construyen (Rollins y Greenwald, 2013; Schneider et al., 2022). Algunos trabajos apuntan a que, en situaciones de interacción triádica con objetos, prevalece una co-regulación simétrica entre ambos participantes (Aureli et al., 2017) que no se observa en condiciones de interacción diádica (i.e., sin objetos), en las que es el adulto quien tiende a atraer, mantener y redirigir la atención del bebé. Asimismo, determinadas rutinas como la lectura compartida parecen favorecer la participación activa y dinámica de adultos y bebés (Rossmanith et al., 2014). Aunque es cierto que ambos parten de diferentes niveles de atención y coordinación, sus intercambios son cada vez más fluidos y sofisticados, lo que apoya el progresivo aumento de episodios de exploración individual del objeto por parte del niño, así como de iniciativas sociales a partir de sus primeros usos convencionales. En ello influyen, además, factores como la sintonía afectiva (Legerstee et al., 2007; Rollins y Greenwald, 2013). Esto,

unido a que el bebé combina de forma cada vez más eficaz diversos recursos comunicativos, parece aumentar la probabilidad de respuesta del adulto, quien posiblemente identifique más fácilmente las acciones del niño como "algo a lo que reaccionar" (Moreno-Núñez, Murillo et al., 2021). Asimismo, en los últimos años diversos trabajos se han interesado por cómo el nivel socioeconómico familiar podría relacionarse con diferentes concepciones y objetivos socializadores de las familias, que podrían influir en el grado de iniciativa y agencialidad interactiva de los bebés (Abels, 2020; Abels y Hutman, 2015) y en el desarrollo de habilidades de coordinación triádica (Gago-Galvagno y Elgier, 2020; Simaes et al., 2022).

3.2.4 Las interacciones triádicas como marco de aprendizaje compartido

Además de sus implicaciones para analizar cómo y cuándo surgen las primeras conductas intencionales en el niño, el estudio de las interacciones triádicas podría ayudar a comprender otros hitos importantes del desarrollo y su naturaleza social. Por ejemplo, desde los seis meses, el número de episodios de interacción triádica en los que participan los niños se relaciona positivamente con su respuesta anticipatoria a la acción del adulto (Brandone et al., 2019), evidencia de una progresiva identificación de los demás como agentes intencionales. Investigaciones recientes, sin embargo, proponen redefinir y abordar el desarrollo de la comprensión intencional desde perspectivas enactivas y corporeizadas, que difieren del paradigma cognitivista clásico: "Se busca atender al aspecto práctico de la cognición social, es decir, qué es lo que hacen los sujetos cuando comprenden intencionalmente a los demás" (Vietri et al., 2021, p. 4). De acuerdo con estos posicionamientos, la propia actividad corporal de los bebés y su congruencia con la acción del adulto constituiría una forma básica de comprensión intencional. Los ajustes corporales anticipatorios de los bebés durante rutinas cotidianas, por ejemplo, cuando en el momento de la comida se inclinan hacia delante y abren la boca para facilitar la acción deliberada de los adultos con la cuchara, parecen apuntar a que "comprenden", desde muy pronto, ciertas intenciones en la acción de los demás. Ello sienta las bases para el origen de la intencionalidad comunicativa en el niño, a partir de las primeras referencias compartidas con otros.

En este proceso, las interacciones triádicas son un importante escenario para el origen y desarrollo de diversos mediadores comunicativos, como los gestos. En este sentido, el gesto de señalar se ha considerado tradicionalmente uno de los primeros hitos comunicativos ya que permiten, hacia el final del primer año, interactuar con los demás y comunicar intenciones, sentimientos y peticiones acerca de referentes presentes, pero distantes. No obstante, algunos estudios incluidos en esta revisión cuestionan que el establecimiento de la referencia compartida se asocie únicamente al gesto de señalar, sugiriendo que los gestos ostensivos (e.g., dar y mostrar) también permiten compartir con el otro el interés por un objeto, por lo que podrían contribuir a la comprensión del referente por parte del niño (Boundy et al., 2016; Guevara et al., 2020; Moreno-Núñez et al., 2020). En los gestos ostensivos el referente ocupa la propia mano y, por tanto, presentan menor complejidad semiótica que los gestos distales. A menudo los niños se dirigen estos gestos a sí mismos (Dupertuis y Moro, 2016), en una autopresentación del objeto con funciones exploratorias y gradualmente reflexivas. La transición entre la exploración individual del objeto y la aparición de conductas comunicativas dirigidas a los demás tendría lugar en un marco triádico, que permite incrementar la complejidad semiótica de la interacción a partir del distanciamiento gradual del referente y la construcción de significados socio-culturales en torno a los usos de los objetos (Moreno-Núñez et al., 2020).

Precisamente, en la interacción con los bebés, los adultos se apoyan en repertorios de acciones diversos que incluyen cómo actúan con los objetos (Bialek et al., 2014). Así, los niños reciben una gran cantidad de información acerca de su entorno mediada, por ejemplo, por la incorporación de elementos y

dinámicas musicales que organizan la interacción y contribuyen a atraer la atención e interés del niño (Moreno-Núñez et al., 2015). Esto promueve el establecimiento de nichos compartidos de actividad y favorece la sintonía afectiva y comunicativa con el adulto. Estas propuestas han dado lugar a nuevos diseños metodológicos basados en la combinación de softwares y procedimientos de análisis que, a partir de los recursos musicales presentes en la interacción, permiten detectar patrones y variaciones a lo largo del tiempo (Alessandroni et al., 2020). Si bien el acompañamiento del adulto se reajusta a medida que el niño gana en agencialidad, sigue ejerciendo de apoyo en la adquisición de habilidades posteriores como evocar eventos u objetos que no están presentes (Palacios et al., 2016, 2018; Palacios y Rodríguez, 2015) o el lenguaje (West e Iverson, 2017). Con relación a esto último, algunos estudios han observado que las emisiones verbales que realizan los adultos durante los períodos manipulativos del niño contienen una mayor proporción de etiquetas, lo que facilita la asociación objeto-palabra y fomenta la interacción a partir de propuestas de acción específicas (por ejemplo, mientras juega con un teléfono y comenta: "¿Estás hablando con papá? Dile hola a papá") (West e Iverson, 2017, p. 198).

Por último, cabe mencionar que a pesar de que son muchos los trabajos que describen estos procesos en el contexto familiar, la literatura reciente apunta a que la sofisticación de las interacciones mediadas por objetos podría trascender este ámbito. De acuerdo con Cárdenas et al. (2020), la mediación del adulto en la escuela infantil podría suponer importantes beneficios para el desarrollo comunicativo a través, por ejemplo, de la lectura compartida de cuentos. Concretamente observaron que, en el grupo experimental, cuya educadora había recibido una formación específica, los bebés llegaban a utilizar más mediadores comunicativos (una combinación de gestos, vocalizaciones y palabras) y usos del objeto que los observados en el grupo control. Ello apunta a que el acompañamiento del adulto durante los encuentros triádicos con el objeto podría apoyar el aprendizaje de habilidades comunicativas en la primera infancia, incluidas las primeras palabras.

4 Discusión y conclusiones

La literatura científica sobre el desarrollo socio-comunicativo temprano ha abordado de forma amplia el estudio de las primeras interacciones triádicas de los bebés con adultos y objetos de su entorno, dando lugar a distintas perspectivas teóricas y metodológicas para su estudio empírico. Este trabajo parte del reconocimiento de esa diversidad de enfoques para analizar algunas cuestiones relevantes sobre las que todavía no hay consenso. Para ello, se ha analizado la producción científica reciente describiendo sus características metodológicas y discutiendo sus resultados en base a un análisis temático. Con respecto a la distribución de los estudios en la muestra, hemos podido comprobar que la mayoría de los estudios se han llevado a cabo en regiones occidentales, principalmente Europa y Norteamérica, reproduciendo así un sesgo habitual en la investigación psicológica a partir del estudio de muestras poblacionales WEIRD (i.e., occidentales, educadas, industrializadas, ricas y democráticas). Desde una perspectiva de género, aunque no es el centro de este estudio, llama la atención que en la mayoría de los estudios de nuestra muestra (excepto en aquellos que no reportan datos sobre el género de los participantes) los datos de los adultos procedían de participantes femeninas, incluidas madres, educadoras e investigadoras. Así pues, consideramos que para obtener una imagen real del desarrollo socio comunicativo temprano en las rutinas cotidianas, sería interesante que futuras investigaciones exploraran las razones que subyacen a este desequilibrio, así como que promuevan la participación de adultos varones en los estudios de interacción con niños pequeños.

Además, el laboratorio ha sido el contexto predominante para la recogida de datos, aunque se observa una tendencia cada vez mayor a desarrollar investigaciones en contextos naturales de desarrollo, como por ejemplo el hogar. En este sentido, los avances tecnológicos de las últimas décadas han derivado en el empleo de instrumentos de registro de la actividad que impactan en el grado de validez ecológica de

los datos. Por ejemplo, algunos estudios utilizan sistemas de cámaras y eye-tracker montados en la cabeza del niño, que permiten registrar el campo de visión de los bebés y la trayectoria visual que siguen al desarrollar una determinada actividad (Yu y Smith, 2013, 2016). Sin embargo, no hemos encontrado estudios que analicen las interacciones triádicas a partir de sensores *wearables*, que podrían añadir información precisa y relevante acerca de la actividad motriz y/o autonomía de los bebés al interactuar con los demás y el entorno (De Barbaro, 2019).

En cuanto a las características metodológicas de los estudios en nuestra muestra, existe una mayor representación de estudios descriptivos frente a los diseños de corte experimental, los cuales son principalmente longitudinales y transversales. Consideramos esto un factor importante, en tanto las investigaciones que abordan los procesos de cambio a lo largo del tiempo permiten una mejor definición de los patrones evolutivos que caracterizan a los fenómenos estudiados (Grimm et al., 2017), especialmente a edades tempranas. Por último, hemos podido constatar cómo, en esta muestra, el uso de estrategias cuantitativas para el análisis de datos prevalece por encima de abordajes cualitativos o mixtos. No obstante, en el marco de los estudios cualitativos, cabe destacar que algunas técnicas utilizadas podrían ser especialmente útiles en el estudio de las interacciones entre adultos y bebés. Por ejemplo, los abordajes microgenéticos permiten realizar análisis exhaustivos y sistemáticos sobre los sutiles cambios que se suceden a pequeña escala en desarrollo temprano (Alessandroni et al., 2020; Lourenço et al., 2021). Asimismo, los estudios etnográficos como el de Abels (2020), enriquecen los datos observacionales a través de anotaciones manuales de las investigadoras durante el acompañamiento continuo de adultos y bebés en sus actividades cotidianas.

Más tarde, el análisis temático de los resultados nos ha permitido identificar distintos dilemas sobre la naturaleza de las primeras experiencias de los bebés con adultos y objetos. Primero, las investigaciones sobre atención conjunta y, específicamente, sobre trayectorias visuales, han permitido aproximarnos a los orígenes de la comunicación referencial entre adulto y niño (Parise et al., 2007; Perra y Gattis, 2012, Tremblay y Rovira, 2007; Yu y Smith, 2013, 2016, 2017). Incluso cuando el bebé está limitado por constricciones biológicas que le impiden sostener la mirada prolongadamente, el seguimiento de la mirada del otro permite compartir referencias sobre el mundo que les rodea (Cleveland y Striano, 2007; Flom et al., 2004; Osório et al., 2011). Sin embargo, los trabajos más recientes consideran que la atención conjunta va más allá de una simple coincidencia de la mirada de dos participantes sobre el mismo objeto: durante la interacción deben, además, estar atentos a los comportamientos del interlocutor y acomodarse a ellos, lo que implica entenderse a sí mismos y al otro como agentes intencionales (Tomasello, 1995). Por ejemplo, el adulto aprovecha los momentos de atención del niño para prolongar los intercambios sirviéndose de diversos mediadores, como la expresión facial o el lenguaje. Esto ha conducido a que cada vez se otorgue un papel más relevante a la exploración y la interacción comunicativa, replanteando el estudio de los fenómenos asociados a los inicios de la triadicidad a partir de las posibilidades que brinda la materialidad (Gibson, 1979). De acuerdo con ello, percepción (atención) y acción forman parte de un mismo proceso de construcción activa del conocimiento (Heras Escribano, 2012; Reddy, 2005), en el que los encuentros atencionales constituirían la base sobre la que establecer instancias de comunicación y acción conjunta con otros. Estas tienen lugar habitualmente a través de los objetos y sus significados culturales (Alessandroni, 2023; Moreno-Núñez, Murillo et al., 2021), promoviendo aprendizajes que, gradualmente, permitirán al niño operar de forma coherente con el entorno socio-material que les rodea (Bruner, 1983; Carpenter et al., 1998; Rogoff, 1990).

Esto nos ha llevado a analizar otros dilemas que rodean al origen de las primeras conductas triádicas, como es a partir de qué momento emergen en el desarrollo del niño. En este sentido, numerosos estudios coinciden en que las habilidades socio-comunicativas de los bebés se sofistican durante el último tercio del primer año de vida (Tomasello et al., 2005; Trevarthen y Hubley, 1978), por lo que era esperable una mayor representación de trabajos centrados en este periodo del desarrollo del niño (Abels, 2020; Abels y Hutman, 2015; Flom et al., 2004; Mendive et al., 2013; Osório et al., 2011; Parise et al., 2007). A pesar de ello, parece poco probable que la intencionalidad comunicativa de los bebés se origine de forma súbita, sino que podría fundamentarse en los episodios interactivos tempranos que los adultos organizan en torno al objeto (Striano y Bertin, 2005; Tremblay y Rovira, 2007). De acuerdo con ello, la coordinación de los procesos necesarios para la interacción triádica comenzaría mucho antes de los nueve meses a partir de la acción del adulto (Amano et al., 2004; Cleveland y Striano, 2007) y de cierta sensibilidad hacia sus señales comunicativas por parte del niño, como el intercambio de miradas sobre los demás y el objeto (Striano y Stahl, 2005). Consecuentemente, antes de que el bebé sea capaz de participar activa y coordinadamente de los intercambios triádicos, el adulto promueve su participación en un proceso continuo de triadicidad temprana (Rodríguez y Moro, 1999) en el que involucra diversos mediadores semióticos. Por un lado, suelen recurrir a acciones reiterativas que favorecen la predictibilidad de los intercambios y, con el tiempo, que adultos y bebés sean capaces de «leer» mutuamente las intenciones del otro (Alessandroni et al., 2020). Por otro lado, la acción mediadora del adulto recae frecuentemente en el uso de objetos y da lugar, a medida que el bebé dispone de mayores herramientas comunicativas, a acciones acordes a las conductas del otro y la funcionalidad cultural de los objetos (Cárdenas et al., 2020; Guevara et al., 2020; Moreno-Núñez et al., 2015, 2017; Rossmanith et al., 2014). Así, alineado con una propuesta socio-constructivista y externalista del desarrollo, el niño construye un «fondo de conocimiento» compartido, que facilita el aprendizaje de numerosas habilidades (por ejemplo, la comprensión intencional) y le habilita para comportarse de forma ajustada a un contexto situado culturalmente (Barresi y Moore, 1996; Brandone et al., 2019, 2020; Rochat, 2007).

No obstante, estos resultados deben considerarse a la luz de algunas limitaciones. Por un lado, la infrarrepresentación de estudios desarrollados en determinados entornos ecológicos cotidianos para el niño (por ejemplo, en escuelas infantiles) y poblaciones más allá del contexto occidental, supone un sesgo que impide generalizar algunos de los resultados en nuestra muestra. Futuras investigaciones dirigidas a comprender de forma global los procesos comunicativos tempranos (Amir y McAuliffe, 2020) deberían aproximarse a una perspectiva ecológica del desarrollo, que atienda a las circunstancias sociales, materiales y culturales cotidianas en las que las interacciones triádicas tienen lugar (Abels, 2020; Keller et al., 2009; Rowe y Goldin-Meadow, 2009). Asimismo, la diversidad de interpretaciones teóricas y conceptuales en torno al origen y desarrollo de las interacciones triádicas ha hecho necesario adoptar un carácter cualitativo a esta revisión, dirigido a describir y discutir los distintos posicionamientos. No obstante, una revisión de tipo cuantitativo (metaanálisis) permitiría continuar explorando alguna de las preguntas específicas que surgen de este trabajo como, por ejemplo, en qué medida las interacciones triádicas benefician el desarrollo comunicativo temprano, o cuál es la relación entre diferentes factores y las experiencias de interacción triádica de los niños. Por ejemplo, el nivel socioeconómico familiar podría asociarse con el grado de exposición a situaciones de interacción triádica (Gago-Galvagno y Elgier, 2020), al igual que las trayectorias de desarrollo atípicas, como la prematuridad al nacimiento, el síndrome de Down o el alto riesgo de presentar TEA, podrían asociarse con dificultades de participación social (Adamson et al., 2009; Benassi et al., 2016; Chiang et al., 2008; Vilaseca et al., 2022), que podrían mejorar tras la intervención temprana (Bejarano-Martín et al., 2022; Mattie y Fanta, 2023; Olafsen et al., 2006).

Un mayor estudio del desarrollo de las interacciones triádicas tempranas (i.e., desde el nacimiento) podría, además, informar posibles mejoras en las prácticas de atención y cuidado de la primera infancia. Por ejemplo, en el entorno familiar, algunos estudios apuntan a que las primeras conductas comunicativas de los bebés no resultan siempre eficaces para suscitar respuestas en los adultos (Boundy et al., 2016; Dimitrova et al., 2015). Por ello, explorar en profundidad el desarrollo dinámico de las primeras señales cognitivas, sociales y comunicativas de los bebés en el marco de las interacciones tempranas en torno a la materialidad podría derivar en la promoción de actividades cotidianas de bajo coste, como la interacción con objetos cotidianos o la lectura compartida de cuentos (Black et al., 2017). Esto podría ser también relevante para explorar el impacto de la salud mental de los padres en el desarrollo de las interacciones triádicas tempranas (por ejemplo, en casos de depresión posparto o estrés parental), ya que la regulación emocional de los padres podría afectar a sus estilos interactivos y comunicativos (Apter y Devouche, 2019). En este sentido, las intervenciones dirigidas a acompañar y facilitar el desarrollo de habilidades interactivas entre padres e hijos a través de video-feedback (Fisher et al., 2016) han demostrado impulsar la autoeficacia percibida por los cuidadores y contribuir a mejorar la salud mental parental (Izett et al., 2021). Además, han demostrado beneficiar el desarrollo infantil, por ejemplo, fomentando las habilidades comunicativas a nivel expresivo y receptivo (Imhof et al., 2023) o reduciendo problemas de conducta (Liu et al., 2021).

Asimismo, dada la trascendencia de los primeros años de vida en el desarrollo psicológico y la importancia de la escuela como contexto socializador, los hallazgos sintetizados en esta revisión podrían resultar pertinentes para los profesionales de educación infantil, en tanto podrían permear los procesos educativos para promover la participación de los bebés con los demás y su entorno. Ello requiere una mayor transferencia entre investigación y escuela, a partir de procesos de reflexión conjunta entre profesionales sobre la propia práctica educativa. Al igual que se observa con los padres y madres en el hogar, las educadoras median frecuentemente la participación de los niños en actividades compartidas cargadas de significados y herramientas socioculturales (Brossard, 2001; Rodríguez y De los Reyes., 2021). Esto tiene lugar, de nuevo, desde un prisma multimodal en el que habitualmente se involucran objetos, materiales, lenguaje, gestos y expresiones emocionales (Estrada, 2021). Si bien este tipo de interacciones forman parte del día a día habitual de los niños, resulta sorprendente la limitada literatura científica que, hasta la fecha, ha explorado las prácticas educativas características de las primeras etapas (0-3 años), en particular acerca del papel que desempeña la cultura material en la configuración del desarrollo temprano en diversos contextos.

En definitiva, los resultados obtenidos en esta revisión permiten concluir que las interacciones triádicas tempranas: (1) se caracterizan por ser intercambios comunicativos multimodales estructurados en secuencias de atención y acción conjuntas a través de la materialidad, (2) se construyen desde los primeros meses de vida y se desarrollan gracias a la mediación del adulto, y (3) constituyen un contexto privilegiado para la construcción de significados socioculturales, fundamentales para la vinculación del bebé con los demás y su entorno.

5 Referencias

Abels, M. (2020). Triadic interaction and gestural communication: Hierarchical and child-centered interactions of rural and urban Gujarati (Indian) caregivers and 9-month-old infants. *Developmental psychology*, *56*(10), 1817–1828. https://doi.org/10.1037/dev0001094

Abels, M., y Hutman, T. (2015). Infants' behavioral styles in joint attention situations and parents' socio-economic status. *Infant Behavior & Development*, *40*, 139–150. https://doi.org/10.1016/j.infbeh.2015.05.004

Adamson, L. B., Bakeman, R., Deckner, D. F., y Romski, M. (2009). Joint engagement and the emergence of language in children with autism and Down syndrome. *Journal of Autism and Developmental Disorders*, *39*(1), 84–96. https://doi.org/10.1007/s10803-008-0601-7

Alessandroni, N. (2023). The road to conventional tool use: Developmental changes in children's material engagement with artifacts in nursery school. *Infancy*, *28*(2), 388–409. https://doi.org/10.1111/infa.12522

Alessandroni, N., Moreno-Núñez, A., Rodríguez, C., y Del Olmo, M. J. (2020). Musical dynamics in early triadic interactions: A case study. *Psychological research*, *84*(6), 1555–1571. https://doi.org/10.1007/s00426-019-01168-4

Amano, S., Kezuka, E., y Yamamoto, A. (2004). Infant shifting attention from an adult's face to an adult's hand: A precursor of joint attention. *Infant Behavior & Development*, *27*(1), 64–80. https://doi.org/10.1016/j.infbeh.2003.06.005

Amir, D. y McAuliffe, K. (2020). Cross-cultural, developmental psychology: Integrating approaches and key insights. *Evolution and Human Behavior*, *41*, 430-444. https://doi.org/10.1016/j.evolhumbehav.2020.06.006

Apter, G. y Devouche, E. (2019). Parental psychiatric disorders and early interaction: Dysregulation and repair. In G. Apter, E. Devouche y M. Gratier (Eds.), *Early interaction and developmental psychopathology* (pp. 107-122). Springer.

Aureli, T., Presaghi, F., y Garito, M. C. (2017). Mother–infant co-regulation in dyadic and triadic contexts at 4 and 6 months of age. *Infant and Child Development*, *27*(3), e2072. https://doi.org/10.1002/icd.2072

Bakeman, R., y Adamson, L. B. (1984). Coordinating attention to people and objects in mother-infant and peer-infant interaction. *Child Development*, *55*(4), 1278-1289.

Barresi, J., y Moore, C. (1996). Intentional relations and social understanding. *Behavioral and Brain Sciences*, *19*(1), 107–154. https://doi.org/10.1017/S0140525X00041790

Bates, E., Camaioni, L., y Volterra, V. (1975). The acquisition of performatives prior to speech. *Merrill-Palmer Quarterly of Behavior and Development*, *21*(3), 205–226.

Bejarano-Martín, Á., Canal-Bedia, R., Magán-Maganto, M., Hernández Fabián, A., Calvarro Castañeda, A. L., Manso de Dios, S., Malmierca García, P., Díez Villoria, E., Jenaro Río, C., y Posada de la Paz, M. (2022). Effect of a focused social and communication intervention on preterm children with ASD: a pilot study. *Journal of autism and developmental disorders*, *52*(4), 1725–1740. https://doi.org/10.1007/s10803-021-05068-8

Benassi, E., Savini, S., Iverson, J. M., Guarini, A., Caselli, M. C., Alessandroni, R., Faldella, G., y Sansavini, A. (2016). Early communicative behaviors and their relationship to motor skills in extremely

preterm infants. *Research in developmental disabilities*, *48*, 132–144. https://doi.org/10.1016/j.ridd.2015.10.017

Bialek, A., Białecka-Pikul, M. y Stępień-Nycz, M. (2014). The nature of child-adult interaction. From turn-taking to understanding pointing and use of pointing gestures. *Psychology of Language and Communication*, *18*(2), 87-105. https://doi.org/10.2478/plc-2014-0008

Black, M. M., Walker, S. P., Fernald, L. C. H., Andersen, C. T., DiGirolamo, A. M., Lu, C., McCoy, D. C., Fink, G., Shawar, Y. R., Shiffman, J., Devercelli, A. E., Wodon, Q. T., Vargas-Barón, E., Grantham-McGregor, S., y Lancet Early Childhood Development Series Steering Committee (2017). Early childhood development coming of age: science through the life course. *Lancet*, *389*(10064), 77–90. https://doi.org/10.1016/S0140-6736(16)31389-7

Boundy, L., Cameron-Faulkner, T., y Theakston, A. (2016). Exploring early communicative behaviours: A fine-grained analysis of infant shows and gives. *Infant Behavior & Development*, *44*, 86–97. https://doi.org/10.1016/j.infbeh.2016.06.005

Brandone, A. C., Stout, W., y Moty, K. (2019). Triadic interactions support infants' emerging understanding of intentional actions. *Developmental science*, *23*(2), e12880. https://doi.org/10.1111/desc.12880

Brandone, A. C., Stout, W., y Moty, K. (2020). Intentional action processing across the transition to crawling: Does the experience of self-locomotion impact infants' understanding of intentional actions? *Infant Behavior & Development*, *60*, 101470. https://doi.org/10.1016/j.infbeh.2020.101470

Braun, V., y Clarke, V. (2006). Using thematic analysis in psychology. *Qualitative Research in Psychology*, *3*(2), 77-101. https://doi.org/10.1191/1478088706qp063oa

Brossard, M. (2001). Situationnes et formes d'apprentissage. Revue suisse des sciences de l'education. *Thema: Eclairage sur la cognition située et modélisations des contextes d'apprentissage*, *23*(3), 423-439. https://doi.org/10.25656/01:3773

Bruner, J. (1983). *Child's talk: Learning to use language*. Norton.C

Burner, T., y Svendsen, B. (2020). Activity Theory—Lev Vygotsky, Aleksei Leont'ev, Yrjö Engeström. En B. Akpan y T. J. Kennedy (Eds.), *Science education in theory and practice. An introductory guide to learning theory* (pp. 311-322). Springer. https://doi.org/10.1007/978-3-030-43620-9_21

Butterworth, G. (1991). The ontogeny and phylogeny of joint visual attention. En A. Whiten (Ed.), *Natural theories of mind: Evolution, development and simulation of everyday mindreading* (pp. 223–232). Basil Blackwell.

Butterworth, G., y Jarrett, N. (1991). What minds have in common is space: Spatial mechanisms serving joint visual attention in infancy. *British Journal of Developmental Psychology*, *9*(1), 55–72. https://doi.org/10.1111/j.2044-835X.1991.tb00862.x

Cárdenas, K., Moreno-Núñez, A., y Miranda-Zapata, E. (2020). Shared book-reading in early childhood education: Teachers' mediation in children's communicative development. *Frontiers in Psychology*, *11*, 2030. https://doi.org/10.3389/fpsyg.2020.02030

Carpendale, J., Müller, U., Wallbridge, B., Broesch, T., Cameron-Faulkner, T., y Ten Eycke, K. (2021). The development of giving in forms of object exchange: Exploring the roots of communication and

morality in early interaction around objects. *Human Development*, *65*(3), 166-179. https://doi.org/10.1159/000517221

Carpenter, M., Nagell, K., Tomasello, M., Butterworth, G., y Moore, C. (1998). Social cognition, joint attention, and communicative competence from 9 to 15 months of age. *Monographs of the Society for Research in Child Development*, *63*(4), 1-174. https://doi.org/10.2307/1166214

Chiang, C. H., Soong, W. T., Lin, T. L. y Rogers, S. J. (2008). Nonverbal communication skills in young children with autism. *Journal of Autism and Developmental Disorders*, *38*, 1898–1906. https://doi.org/10.1007/s10803-008-0586-2

Cleveland, A., y Striano, T. (2007). The effects of joint attention on object processing in 4- and 9-month-old infants. *Infant Behavior & Development*, *30*(3), 499–504. https://doi.org/10.1016/j.infbeh.2006.10.009

Corkum, V., y Moore, C. (1998). The origins of joint visual attention in infants. *Developmental Psychology*, *34*(1), 28–38. https://doi.org/10.1037/0012-1649.34.1.28

Danis, A., Bourdais, C. y Ruel, J. (2000). The co-construction of joint action between mothers and 2–4-month-old infants: The mother's role. *Infant and Child Development*, *9*(4), 181-198. https://doi.org/10.1002/1522-7219(200012)9:4<181::AID-ICD227>3.0.CO;2-S

De Barbaro, K. (2019). Automated sensing of daily activity: A new lens into development. *Developmental Psychobiology*, *61*(3), 444–464. https://doi.org/10.1002/ dev.21831

De Barbaro, K., Johnson, C. M., Forster, D., y Deák, G. O. (2016). Sensorimotor decoupling contributes to triadic attention: A longitudinal investigation of mother-infant-object interactions. *Child development*, *87*(2), 494–512. https://doi.org/10.1111/cdev.12464

De Barbaro, K., Johnson, C. M., y Deák, G. O. (2013). Twelve-month ‘‘social revolution’’ emerges from mother-infant sensorimotor coordination: A longitudinal investigation. *Human Development*, *56*(4), 223-248. https://doi.org/10.1159/000351313

De Schuymer, L., De Groote, I., Striano, T., Stahl, D., y Roeyers, H. (2011). Dyadic and triadic skills in preterm and full term infants: A longitudinal study in the first year. *Infant Behavior & Development*, *34*(1), 179-188. https://doi.org/10.1016/j.infbeh.2010.12.007

Dimitrova, N., y Moro, C. (2013). Common ground on object use associates with caregivers' gesturese. *Infant Behavior & Development*, *36*(4), 618–626. https://doi.org/10.1016/j.infbeh.2013.06.006

Dimitrova, N., Moro, C., y Mohr, C. (2015). Caregivers interpret infants' early gestures based on shared knowledge about referents. *Infant Behavior & Development*, *39*, 98–106. https://doi.org/10.1016/j.infbeh.2015.02.015

Dupertuis, V., y Moro, C. (2016). Self-Directed ostensions and mediations of the adult at the age of 8-, 12- and 16 months. *Integrative psychological & behavioral science*, *50*(4), 621–633. https://doi.org/10.1007/s12124-016-9350-x

Estrada, L. (2021). Tipos de situaciones educativas en la escuela infantil: el rol mediador de los maestros. En C. Rodríguez y J. L. De los Reyes (Coords.), *Los objetos sí importan: Acción educativa en la escuela infantil* (pp. 159-181). Horsori.

Fisher, P. A., Frenkel, T. I., Noll, L. K., Berry, M., y Yockelson, M. (2016). Promoting healthy child development via a two-generation translational neuroscience framework: The filming interactions to nurture development video coaching program. *Child development perspectives*, *10*(4), 251–256. https://doi.org/10.1111/cdep.12195

Fivaz-Depeursinge, E., y Corboz-Warnery, A. (1999). *The primary triangle: A developmental systems view of mothers, fathers, and infants.* Basic Books.

Flom, R., Deák, G. O., Phill, C. G., y Pick, A. D. (2004). Nine-month-olds' shared visual attention as a function of gesture and object location. *Infant Behavior & Development*, *27*(2), 181–194. https://doi.org/10.1016/j.infbeh.2003.09.007

Fogel, A. (1993). *Developing through relationships: Origins of communication, self, and culture*. University of Chicago Press.

Fogel, A. y Thelen, E. (1987). Development of early expressive and communicative action: Reinterpreting the evidence from a dynamic systems perspective. *Developmental Psychology*, *23*(6), 747-761. https://doi.org/10.1037/0012-1649.23.6.747

Foot, K. A. (2014). Cultural-historical activity theory: Exploring a theory to inform practice and research. J*ournal of Human Behavior in the Social Environment*, *24*(3), 329-347. https://doi.org/10.1080/10911359.2013.831011

Gabouer, A., y Bortfeld, H. (2021). Revisiting how we operationalize joint attention. *Infant Behavior & Development*, *63*, 101566. https://doi.org/10.1016/j.infbeh.2021.101566

Gago-Galvagno, L. G., y Elgier, A. M. (2020). Social and individual factors modulate parent-infant interactions: Lessons from free play sessions in an Argentine sample. *Infant Behavior & Development*, *61*, 101496. https://doi.org/10.1016/j.infbeh.2020.101496

Gattis, M., Winstanley, A., Sperotto, R., Putnick, D. L., y Bornstein, M. H. (2020). Foundations of attention sharing: Orienting and responding to attention in term and preterm 5-month-old infants. *Infant Behavior & Development*, *61*, 101466. https://doi.org/10.1016/j.infbeh.2020.101466

Gibson, J. J. (1979). *The Ecological Approach to Visual Perception.* Houghton Mifflin.

Grimm, K. J., Davoudzadeh, P., y Ram, N. (2017). Developments in the analysis of longitudinal data. *Monographs of the Society for Research in Child Development*, *82*(2), 46–66. https://doi.org/10.1111/mono.12298.

Guevara, I., Moreno-Llanos, I. y Rodríguez, C. (2020). The emergence of gestures in the first year of life in the Infant School classroom. *European Journal of Psychology of Education*, *35*, 265–287. https://doi.org/10.1007/s10212-019-00444-6

Heras Escribano, M. (2012). Comprender la realidad sin representaciones: Affordances y psicología ecológica. *Ciencia Cognitiva*, *6*(2), 48-50.

Hobson, R. P., Patrick, M. P., Crandell, L. E., García Pérez, R. M., y Lee, A. (2004). Maternal sensitivity and infant triadic communication. *Journal of child psychology and psychiatry, and allied disciplines*, *45*(3), 470–480. https://doi.org/10.1111/j.1469-7610.2004.00238.x

Imhof, A., Liu, S., Schlueter, L., Phu, T., Watamura, S., y Fisher, P. (2023). Improving children’s expressive language and auditory comprehension through responsive caregiving: Evidence from a randomized controlled trial of a strength-based video-coaching intervention. *Prevention Science*, *24*, 84–93. https://doi.org/10.1007/s11121-022-01455-4

Izett, E., Rooney, R., Prescott, S. L., De Palma, M., y McDevitt, M. (2021). Prevention of mental health difficulties for children aged 0–3 years: A review. *Frontiers in Psychology*, *11,* 500361. https://doi.org/10.3389/fpsyg.2020.500361

Jacobson, S. y Degotardi, S. (2022). A dynamic systems approach to joint attention in an infant-toddler early childhood centre. *Learning, Culture and Social Interaction*, *32*, 100574. https://doi.org/10.1016/j.lcsi.2021.100574

Keller, H., Borke, J., Staufenbiel, T., Yovsi, R. D., Abels, M., Papaligoura, Z., Jensen, H., Lohaus, A., Chaudhary, N., Lo, W., y Su, Y. (2009). Distal and proximal parenting as alternative parenting strategies during infants' early months of life: A cross-cultural study. *International Journal of Behavioral Development, 33,* 412–420. https://doi.org/10.1177/0165025409338441

Kmet, L. M., Lee, R. C., y Cook, L. S. (2004). *Standard quality assessment criteria for evaluating primary research papers from a variety of fields*. Alberta Heritage Foundation for Medical Research. https://doi.org/10.7939/R37M04F16

Legerstee, M., Markova, G., y Fisher, T. (2007). The role of maternal affect attunement in dyadic and triadic communication. *Infant Behavior & Development*, *30*(2), 296–306. https://doi.org/10.1016/j.infbeh.2006.10.003

León, M. J. y Olhaberry, M. (2020). Triadic interactions, parental reflective functioning, and early social-emotional difficulties. *Infant mental health journal*, *41*(4), 431–444. https://doi.org/10.1002/imhj.21844

Liu, S., Phu, T., Dominguez, A., Hurwich-Reiss, E., McGee, D., Watamura, S. y Fisher, P. (2021). Improving caregiver self-efficacy and children's behavioral outcomes via a brief strength-based video coaching intervention: Results from a randomized controlled trial. *Prevention Science*. https://doi.org/10.1007/s11121-021-01251-6

Lourenço, V., Coutinho, J., y Pereira, A. F. (2021). Advances in microanalysis: Magnifying the social microscope on mother-infant interactions. *Infant Behavior & Development*, *64*, 101571. https://doi.org/10.1016/j.infbeh.2021.10157

Mattie, L. J., y Fanta, D. (2023). Joint engagement and early language abilities in young children with Down syndrome. *Frontiers in Psychology*, *14,* 1152559. https://doi.org/10.3389/fpsyg.2023.1152559

McCune, L. y Zlatev, J. (2015). Dynamic systems in semiotic development: The transition to reference. *Cognitive Development*, *36*, 161-170. https://doi.org/10.1016/j.cogdev.2015.09.010

Mendive, S., Bornstein, M. H., y Sebastián, C. (2013). The role of maternal attention-directing strategies in 9-month-old infants attaining joint engagement. *Infant Behavior & Development*, *36*(1), 115–123. https://doi.org/10.1016/j.infbeh.2012.10.002

Moher, D., Liberati, A., Tetzlaff, J., Altman, D. G., The PRISMA Group. (2009). Preferred reporting items for systematic reviews and meta-analyses: The PRISMA Statement. *PLoS Medicine*, *6*(7), e1000097. https://doi.org/10.1371/journal.pmed.1000097

Montero, I. y León, O. G. (2002). Clasificación y descripción de las metodologías de investigación en Psicología. *Revista Internacional de Psicología Clínica y de la Salud*, *2*(3), 503-508.

Moreno-Núñez, A., Fernández-Alcaide, A., y Martín-Ruiz, N. (2021). El desarrollo de las interacciones triádicas tempranas: Dinámicas musicales de interacción en el primer año. *Revista de Psicología*, *088*. https://dx.doi.org/10.24215/2422572Xe088

Moreno-Núñez, A., Murillo, E., Casla, M., y Rujas, I. (2021). The multimodality of infant's rhythmic movements as a modulator of the interaction with their caregivers. *Infant Behavior & Development*, *65*, 101645. https://doi.org/10.1016/j.infbeh.2021.101645

Moreno-Núñez, A., Rodríguez, C., y Del Olmo, M. J. (2015). The rhythmic, sonorous and melodic components of adult-child-object interactions between 2 and 6 months old. *Integrative psychological & behavioral science*, *49*(4), 737–756. https://doi.org/10.1007/s12124-015-9298-2

Moreno-Núñez, A., Rodríguez, C., y Del Olmo, M. J. (2017). Rhythmic ostensive gestures: How adults facilitate infants' entrance into early triadic interactions. *Infant Behavior & Development*, *49*, 168-181. https://doi.org/10.1016/j.infbeh.2017.09.003

Moreno-Núñez, A., Rodríguez, C., y Miranda-Zapata, E. (2020). Getting away from the point: the emergence of ostensive gestures and their functions. *Journal of child language*, *47*(3), 556–578. https://doi.org/10.1017/S0305000919000606

Olafsen, K. S., Rønning, J. A., Kaaresen, P. I., Ulvund, S. E., Handegård, B. H., y Dahl, L. B. (2006). Joint attention in term and preterm infants at 12 months corrected age: the significance of gender and intervention based on a randomized controlled trial. *Infant Behavior & Development*, *29*(4), 554–563. https://doi.org/10.1016/j.infbeh.2006.07.004

Osório, A., Martins, C., Meins, E., Martins, E. C., y Soares, I. (2011). Individual and relational contributions to parallel and joint attention in infancy. *Infant Behavior & Development*, *34*(4), 515–524. https://doi.org/10.1016/j.infbeh.2011.07.005

Page, M. J., Moher, D., Bossuyt, P. M., Boutron, I., Hoffmann, T. C., Mulrow, C. D., Shamseer, L., Tetzlaff, J. M., Akl, E. A., Brennan, S. E., Chou, R., Glanville, J., Grimshaw, J. M., Hróbjartsson, A., Lalu, M. M., Li, T., Loder, E. W., Mayo-Wilson, E., McDonald, S., ... McKenzie, J. E. (2021). PRISMA 2020 explanation and elaboration: Updated guidance and exemplars for reporting systematic reviews. *British Medical Journal*, *372*, n160. https://doi.org/10.1136/bmj.n160

Palacios, P. y Rodríguez, C. (2015). The development of symbolic uses of objects in infants in a triadic context: A pragmatic and semiotic perspective. *Infant and Child Development*, *24*(1), 23-43. https://doi.org/10.1002/icd.1873

Palacios, P., Rodríguez, C., y Méndez-Sánchez, C. (2018). Communicative mediation by adults in the construction of symbolic uses by infants. *Integrative psychological & behavioral science*, *52*(2), 209–227. https://doi.org/10.1007/s12124-018-9422-1

Palacios, P., Rodríguez, C., Méndez-Sánchez, C., Hermosillo-De-La-Torre, A.-E., Sahagún, M.-Á., y Cárdenas, K. (2016). The development of the first symbolic uses in Mexican children from the pragmatics of object. *Estudios de Psicología*, *37*(1), 59–89. https://doi.org/10.1080/02109395.2015.1122437

Parise, E., Cleveland, A., Costabile, A., y Striano, T. (2007). Influence of vocal cues on learning about objects in joint attention contexts. *Infant Behavior & Development*, *30*(2), 380–384. https://doi.org/10.1016/j.infbeh.2006.10.006

Perra, O., y Gattis, M. (2012). Attention engagement in early infancy. *Infant Behavior & Development*, *35*(4), 635–644. https://doi.org/10.1016/j.infbeh.2012.06.004

Rączaszek-Leonardi, J., Rossmanith, N., Nomikou, I., y Rohlfing, K. J. (2019). Levels of coordination in early semantic development. *Psychology of Language and Communication*, *23*(1), 212-237. https://doi.org/10.2478/plc-2019-0010

Reddy, V. (2005). Before the 'Third Element': understanding attention to self. En T. McCormack, C. Hoerl, J. Roessler y N. Eilan (eds.), *Joint Attention: Communication and Other Minds: Issues in*

Philosophy and Psychology (pp. 85-109). Oxford University Press. https://doi.org/10.1093/acprof:oso/9780199245635.003.0005

Reddy, V. (2010). *How infants know minds*. Harvard University Press.

Rochat, P. (2007). Intentional action arises from early reciprocal exchanges. *Acta psychologica*, *124*(1), 8–25. https://doi.org/10.1016/j.actpsy.2006.09.004

Rodríguez, C. (2006). *Del ritmo al símbolo. Los signos en el nacimiento de la inteligencia.* Horsori.

Rodríguez, C. (2012). El adulto como guía: ¿El eslabón perdido de desarrollo temprano? *Padres y Maestros*, *344*, 23-26.

Rodríguez, C. y De los Reyes, J. L. (2021). *Los objetos sí importan. Acción educativa en la escuela infantil.* Horsori.

Rodríguez, C., y Moro, C. (1999). *El mágico número tres: Cuando los niños aún no hablan.* Paidós.

Rogoff, B. (1990). *Apprenticeship in thinking: Cognitive development in social context.* Oxford University Press.

Rollins, P. R., y Greenwald, L. C. (2013). Affect attunement during mother-infant interaction: How specific intensities predict the stability of infants' coordinated joint attention skills. *Imagination, Cognition and Personality*, *32*(4), 339–366. https://doi.org/10.2190/IC.32.4.c

Rossmanith, N., Costall, A., Reichelt, A. F., López, B., y Reddy, V. (2014). Jointly structuring triadic spaces of meaning and action: Book sharing from 3 months on. *Frontiers in Psychology*, *5*. https://doi.org/10.3389/fpsyg.2014.01390

Rowe, M. L., y Goldin-Meadow, S. (2009). Differences in early gesture explain SES disparities in child vocabulary size at school entry. *Science, 323,* 951–953. http://dx.doi.org/10.1126/science.1167025

Schneider, J. L., Roemer, E. J., Northrup, J. B., e Iverson, J. M. (2022). Dynamics of the dyad: How mothers and infants co-construct interaction spaces during object play. *Developmental science*, e13281. https://doi.org/10.1111/desc.13281

Serra, J., Miguel, H., Moura, A. A., Sampaio, A., y Pereira, A. F. (2020). The effect of play task on maternal touch patterns when interacting with their 12 months-old infants: An exploratory study. *Infant Behavior & Development*, *59*, 101438. https://doi.org/10.1016/j.infbeh.2020.101438

Simaes, A. C., Gago-Galvagno, L. G., Passarini, L. A., Trenado, R. M., y Elgier, A. M. (2022). Associations between maternal behavior, infant joint attention, and social vulnerability. *Cognitive development*, *61*, 101141. https://doi.org/10.1016/j.cogdev.2021.101141

Siposova, B. y Carpenter, M. (2019). A new look at joint attention and common knowledge. *Cognition*, *189*, 260-274. https://doi.org/10.1016/j.cognition.2019.03.019

Solovieva, Y., García-Flores, M. A., Moreno-Agundis, M. S., y Quintanar-Rojas, L. (2020). Caracterización de acciones objetales y la producción verbal en la edad preescolar menor. *Psicologia em Estudo*, *25*, e45979. https://doi.org/10.4025/psicolestud.v25i0.45979

Solovieva, Y., y Quintanar, L. (2021). The first year developmental crisis: Origin of cultural action. *Frontiers in Psychology*, *12*, 686761. https://doi.org/10.3389/fpsyg.2021.686761

Striano, T., y Bertin, E. (2005). Social-cognitive skills between 5 and 10 months of age. The *British journal of developmental psychology*, *23*(4), 559–568. https://doi.org/10.1348/026151005X26282

Striano, T., y Reid, V. M. (2006). Social cognition in the first year. *Trends in Cognitive Sciences*, *10*(10), 471–476. https://doi.org/10.1016/j.tics.2006.08.006

Striano, T., y Stahl, D. (2005). Sensitivity to triadic attention in early infancy. *Developmental science*, *8*(4), 333–343. https://doi.org/10.1111/j.1467-7687.2005.00421.x

Striano, T., Stahl, D., Cleveland, A., y Hoehl, S. (2007). Sensitivity to triadic attention between 6 weeks and 3 months of age. *Infant Behavior & Development*, *30*(3), 529–534. https://doi.org/10.1016/j.infbeh.2006.12.010

Suárez-Rivera, C., Smith, L. B., y Yu, C. (2019). Multimodal parent behaviors within joint attention support sustained attention in infants. *Developmental psychology*, *55*(1), 96–109. https://doi.org/10.1037/dev0000628

Thelen, E., y Smith, L. B. (1994). *A dynamic systems approach to the development of cognition and action.* MIT Press.

Tomasello, M. (1995). Joint attention as social cognition. En C. Moore y P. J. Dunham (Eds.), *Joint attention: Its origins and role in development* (pp. 103–130). Lawrence Erlbaum Associates, Inc.

Tomasello, M. (2013). *Los orígenes de la comunicación humana*. Katz.

Tomasello, M., Carpenter, M., Call, J., Behne, T., y Moll, H. (2005). Understanding and sharing intentions: the origins of cultural cognition. *The Behavioral and brain sciences*, *28*(5), 675–735. https://doi.org/10.1017/S0140525X05000129

Tomasello, M. y Farrar, M. J. (1986). Joint attention and early language. *Child Development*, *57*(6), 1454–1463. https://doi.org/10.2307/1130423

Trautman, C. H., y Rollins, P. (2006). Child-centered behaviors of caregivers with 12-month-old infants: Associations with passive joint engagement and later language. *Applied Psycholinguistics*, *27*(3), 447-463. https://doi.org/10.1017/S0142716406060358

Tremblay, H., y Rovira, K. (2007). Joint visual attention and social triangular engagement at 3 and 6 months. *Infant Behavior & Development*, *30*(2), 366–379. https://doi.org/10.1016/j.infbeh.2006.10.004

Trevarthen, C., y Hubley, P. (1978). Secondary intersubjectivity: confidence, confiding, and acts of meaning in the first year. En J. Lock (Ed.), *Action, Gesture and Symbol* (pp. 183-229). Academic Press.

Trevarthen, C. y Reddy, V. (2017). Consciousness in infants. En M. Velmans y S. Schneider (Eds.), *The Blackwell Companion to Consciousness* (2ª ed., pp. 45-62). Blackwell.

Veraksa, N., y Veraksa, A. (2018). Lev Vygotsky's cultural-historical theory of development and the problem of mental tools. *Papeles del Psicólogo*, *39*(2), 150-154. https://doi.org/10.23923/pap.psicol2018.2862

Vietri, M., Alessandroni, N., y Piro, M. C. (2021). Usos de objetos y anticipaciones corporales en el desarrollo temprano. *Revista De Psicología*, *089*. https://doi.org/10.24215/2422572Xe089

Vilaseca, R., Rivero, M., Leiva, D. y Ferrer, F. (2022). Mothers' and fathers' parenting and other family context variables linked to developmental outcomes in young children with intellectual disability: A two-wave longitudinal study. *Journal of Developmental and Physical Disabilities*. https://doi.org/10.1007/s10882-022-09856-7

Vygotsky, L. S. (1932/1996). El primer año. En Vygotsky, L. S., *Obras escogidas IV. Psicología infantil* (pp. 275-318). Visor.

Vygotsky, L. S. (1934/1986). *Thought and language*. MIT Press.

Vygotsky, L. S. (1978). *Mind in society: The development of higher psychological processes.* Harvard University Press.

Vygotsky, L. S. (1984). Aprendizaje y desarrollo intelectual en la edad escolar. *Journal for the Study of Education and Development, Infancia y Aprendizaje*, *27/28*, pp. 105-116. https://doi.org/10.1080/02103702.1984.10822045

West, K. L. e Iverson, J. M. (2017). Language learning is hands-on: Exploring links between infants' object manipulation and verbal input. *Cognitive Development*, *43*, 190-200. https://doi.org/10.1016/j.cogdev.2017.05.004

Yu, C., y Smith, L. B. (2013). Joint attention without gaze following: Human infants and their parents coordinate visual attention to objects through eye-hand coordination. *PLOS ONE*, *8*(11): e79659. https://doi.org/10.1371/journal.pone.0079659

Yu, C. y Smith, L. B. (2016). The social origins of sustained attention in one-year-old human infants. *Current Biology*, *26*, 1235–1240. http://dx.doi.org/10.1016/j.cub.2016.03.026

Yu, C., y Smith, L. B. (2017). Multiple sensory-motor pathways lead to coordinated visual attention. *Cognitive science*, *41*, Suppl 1, 5–31. https://doi.org/10.1111/cogs.12366

Zuccarini, M., Guarini, A., Iverson, J. M., Benassi, E., Savini, S., Alessandroni, R., Faldella, G., y Sansavini, A. (2018). Does early object exploration support gesture and language development in extremely preterm infants and full-term infants? *Journal of communication disorders*, *76*, 91–100. https://doi.org/10.1016/j.jcomdis.2018.09.004

Conflictos de interés

Las autoras declaran que la investigación se llevó a cabo en ausencia de relaciones comerciales o financieras que pudieran interpretarse como un posible conflicto de intereses.

Contribuciones de las autoras

AMG: conceptualización, metodología, gestión de datos, análisis, investigación, redacción del manuscrito original.
AMN: conceptualización, redacción del manuscrito original, revisión y edición, supervisión.

Financiación

Este estudio ha contado con el apoyo de las Ayudas para Contratos Predoctorales para la Formación de Doctores (convocatoria 2020), de las que es beneficiaria la primera autora [referencia PRE2020-094773], y del proyecto PID2019-108845GA-I00/AEI/10.13039/501100011033, co-financiados por la Agencia Estatal de Investigación (Ministerio de Ciencia e Innovación, España) y por el Fondo Social Europeo (FSE).
